# Expanding the Antimicrobial Toolbox with Therapeutic Viruses: Mechanisms, Pharmaceutical Formulation, and Translational Outlook

**DOI:** 10.3390/ph19030478

**Published:** 2026-03-14

**Authors:** Margarita Strimaite, Holly A. Bailey, Diba Keyhanfar, Roxy Lee, Gareth R. Williams

**Affiliations:** 1 UCL Centre for Advanced Biomedical Imaging, University College London, 72 Huntley Street, London WC1E 6DD, UK; 2 UCL School of Pharmacy, University College London, 29-39 Brunswick Square, London WC1N 1AX, UK; diba.keyhanfar.14@ucl.ac.uk; 3 Harper and Keele Veterinary School, Keele University, Keele ST5 5BG, UK; 4 Department of Energy Conversion and Storage, Technical University of Denmark, Fysikvej, Building 310, 2800 Kongens Lyngby, Denmark; roxle@dtu.dk

**Keywords:** antimicrobial resistance, One Health, bacteriophage therapy, virus-based antimicrobials, phage–antibiotic synergy, virophages, mycoviruses, parasite-associated viruses, phage formulation, alternative antimicrobials, translational virotherapy

## Abstract

Infectious diseases continue to represent one of the most persistent challenges in human health and agricultural productivity. These diseases are caused by a wide range of pathogenic microorganisms, including bacteria, fungi, viruses, and parasites. Antimicrobial resistance, or AMR, is the gradual evolution of pathogenic microbes to evade the action of commonly used antimicrobial agents (antibiotics, antifungals, antivirals, and antiparasitics) and is a problem that continues to be exacerbated by the inappropriate use of antimicrobials across multiple global industries. AMR poses a major threat to our society, and without mitigation, will lead to devastating consequences with broad implications beyond human health. The search for alternative or complementary therapies to conventional antimicrobials is, therefore, of the utmost priority. In this review, we first outline the prevalence of AMR and the circumstances driving the proliferation of AMR, which is widely recognised as a One Health issue—through interconnected factors within human and veterinary medicine, agricultural practice, and the environment. We next summarise the various classes of pathogens, common antimicrobial agents, and the mechanisms which pathogens have evolved to evade antimicrobial action. Within this context, we discuss the therapeutic potential of bacteriophages, virophages, and mycoviruses against antimicrobial-resistant infections, and consider the future perspectives of virus-based formulations.

## 1. Introduction

It is difficult to overstate the importance and positive impact to society of the discovery of antibiotics, antifungals, antivirals, and synthetic pesticides in the early to mid-20th century. Millions of human lives have been saved through the revolutionised infection treatment, vastly improved food security, and a substantial rise in quality of life spurred on by these technologies. The use of a variety of different antimicrobial agents is both ubiquitous as well as necessary in modern society—from antiseptics, disinfectants, and prophylactic and therapeutic drugs to prevent and treat infections in humans and animals, to antimicrobial pesticides used to protect yields in agriculture and aquaculture. Though such measures continue to be effective to achieve these aims today, there is a very real possibility that this may no longer be the case in the future, due to the rise of antimicrobial resistance (AMR).

AMR is a natural process, a special case of evolution, whereby microbial populations gradually acquire the ability to resist the effects of antimicrobial agents. Resistance in a specific microbial population may emerge de novo, because of a random genetic mutation which so happens to endow the microbe with a phenotype that is in some way beneficial to survival in the presence of that antimicrobial agent, though it may not necessarily be beneficial or indeed may be detrimental under normal conditions. Microbes within the population which do not have this beneficial mutation will be more likely to die and less likely to proliferate in the presence of that antimicrobial agent (and thus less likely to pass on their genotype). A selection pressure is exerted that results in a higher proportion of the microbial population exhibiting this beneficial mutation over time. Not only do such mutations gradually come to dominate within the originally exposed microbial population, but they may also spread to naïve populations through various gene transfer mechanisms. If these mutated microbes cause an infection, treatment with the initial antimicrobial agent will be rendered ineffective.

**Figure 1:**
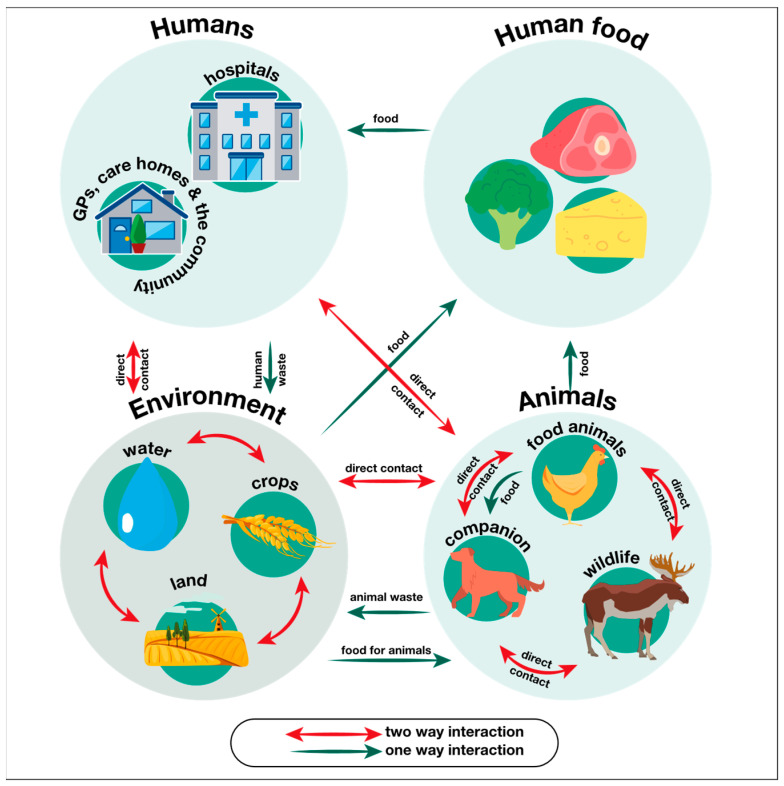
Figure illustrating some of the ‘One Health’ interactions which facilitate the intra- and inter-species spread of resistance genes in microbes. Reproduced from reference [1] under the Open Government License.

From this simplified case, it is apparent that any factor which increases the frequency or duration of microbial exposure to antimicrobial agents will necessarily lead to an increased rate of de novo AMR gene emergence and proliferation. This includes therapeutic use of antimicrobials as well as misuse, such as incorrect or unnecessary prescription (e.g., antibiotics for a viral infection), excessive use (dosage or course length), and poor patient compliance with dosing regimen or course length. Other contributing factors include (but are not limited to) the large-scale prophylactic use of antibiotics in livestock to prevent disease, improper disposal of antibiotics, and large-scale application of pesticides and antibiotics to crops. Factors which affect interactions between different microbial populations will affect the rate of gene transfer between them, and, therefore, the number of separate microbial populations which acquire existing AMR genes. In the globalised world, such factors are inherently more difficult to mitigate against, as there exist myriad opportunities for contact and gene flow between microbial groups (Figure 1).

Factors in the former category may be addressed, at least in part, by measures such as education and awareness campaigns for both the public and healthcare practitioners, minimising the need for antimicrobials by implementing better disease control strategies, antimicrobial stewardship strategies to ensure appropriate and optimal use, legislation, or policy measures to reduce the use of prophylactic antimicrobial treatments in livestock and crops, as well as improving equity of access (technology, diagnostic resources, appropriate antimicrobials, financial resources, etc.).

As the cognisance of the threat of AMR grows, many governments and organisations have produced resources, legislature, and policy commitments to address AMR. In the United Kingdom (UK), for instance, the government published its first AMR Strategy and Action Plan in 2000, and the current 20-year strategy (‘Contained and Controlled’) in 2019. The first 5-year plan within this aimed to address three key themes—reducing the need for and exposure to antimicrobials, optimising their use, and investing in research, development, and improvement in access. Additionally, the English Surveillance Programme for Antimicrobial Utilisation and Resistance (ESPAUR) collects data on the use of antimicrobial agents and the prevalence of resistance, which are published in annual reports and used to inform the development of further national action plans and policies. In the second 5-year plan as part of the UK Governments’ current 20-year vision for AMR, an additional theme was added which centres around AMR diplomacy in a worldwide setting. This is particularly important as even optimally implemented national antimicrobial stewardship efforts can be undermined by continued global circulation of resistance genes, and, therefore, will require a concerted and global effort. AMR diplomacy aims to facilitate international collaboration to develop consistent regulatory standards, enable higher quality, holistic surveillance, and data sharing, as well as to pool resources to support infection prevention and control, diagnostics, and research efforts (particularly to support low- and middle-income countries).

Given that our list of available antimicrobials is finite, and that the development of new antimicrobials is unfortunately very slow [2], it is not inconceivable that the continued spread of AMR without mitigation may eventually result in a world with few or no effective antimicrobial agents. Such an outcome would be catastrophic, with immeasurable human cost and a precipitous decline in quality of life. Medical procedures such as surgeries, cancer treatments, and organ transplantation would become prohibitively risky due to the inability to treat prophylactically as well as in the event of post-operative infection. Routine post-treatment infections for minor procedures such as tooth extractions would, in such a world, become potentially life-threatening. Agriculture and aquaculture productivity would plummet, and food supply chains would be severely disrupted.

One context in which the presence of resistant strains of pathogens is particularly devastating is within the healthcare setting, such as in hospitals or nursing homes. Globally, healthcare-associated infections (also known as nosocomial infections) account for approximately 136 million cases of drug-resistant infections each year [3] and are particularly dangerous as the concentration of vulnerable patients in the resident populations of such facilities is very high (e.g., immunocompromised, elderly, chronically ill, or post-operative patients). The transmission potential is also high, as the patients are often in close proximity to one another, and in frequent contact with staff. Moreover, as the use of antimicrobial drugs and antiseptic/disinfectant agents in these settings is much higher than in the community, the selection pressure exerted upon pathogenic microorganisms is consequently also much higher, and, therefore, the persistence and spread of antimicrobial resistance genes is strongly selected for.

Bacterial resistance alone is projected to directly cause up to 1.91 million annual deaths and contribute to up to 8.22 million annual deaths globally by 2050 (compared to directly causing 1.14 million deaths and contributing to 4.71 million deaths in 2021) [4]. It is estimated that up to 95 million cumulative deaths between 2025 and 2050 may be averted with improvements to care standards (e.g., better access to antibiotics) and a further 11 million deaths could be avoided through prioritising research to develop antibacterial drugs, highlighting the importance of immediate interventive action.

High-quality epidemiological data on mortality mediated by AMR in non-bacterial pathogens (fungi, viruses, and parasites) are not as readily available due to much lower surveillance rates than for bacterial AMR. Invasive fungal diseases, including both drug-susceptible and drug-resistant infections, have been previously estimated to kill approximately 1.5 million people per annum [5], with more recent estimates being closer to 2.5 million deaths caused directly by invasive fungal infections [6]. Resistance to antifungal drugs has been historically considered a less pressing issue than antibacterial resistance, partly due to the later and more limited clinical and agricultural deployment of antifungal agents (compared to antibiotics). Additionally, unlike in bacteria, the dissemination of resistance genes in fungi does not usually proceed by horizontal gene transfer and is thus usually slower and less efficient. These factors meant that multidrug-resistant fungal species emerged relatively late, with C. auris being the first globally recognised outbreak-associated fungal species to demonstrate multi-drug resistance in 2009 [7]. With such high rates of infection and mortality globally, more attention is now shifting towards fungal AMR as an imminent threat.

Antiviral therapies are predominantly used on a long-term basis to manage chronic viral infections (such as for treatment of human immunodeficiency virus (HIV), herpesviruses, and hepatitis B and C), and to a lesser degree as short-term courses to treat acute infections such as influenza. Consequently, antiviral resistance has been most extensively studied in the context of chronic viral infections, with long-term exposure and poor patient adherence exerting selective pressure for the emergence of resistant viral variants. Over 31.6 million patients were using antiretroviral therapy for prophylaxis or suppression of active HIV infections at the end of 2024. One modelling study [8] estimates that acquired resistance to dolutegravir (a first-line antiretroviral drug) may rise from 18.5% in 2023 to 41.7% by 2035, jeopardising the treatment of many millions of patients and exposing them to risk of developing acquired immunodeficiency syndrome.

Similarly, concerns around AMR in parasites are rising, most notably in the treatment of malaria which is caused by protozoan species in the genus *Plasmodium*. Many *Plasmodium* species are now resistant to chloroquine (which used to be a first-line malaria treatment) and other available antimalarial drugs, with resistance now emerging against artemisinin [9], which is the current first-line treatment (though typically used in combination with other antimalarial agents). With an estimated 241 million cases of malaria in 2020 alone [9], it is not difficult to see the potential healthcare ramifications if the rate of treatment failure keeps rising due to AMR.

This review provides a broad, cross-kingdom overview of antimicrobial resistance as a One Health challenge, and of the therapeutic potential of viruses which target the different pathogen classes (bacterial, fungal, viral, and parasitic). In the first part of this review, we present a comprehensive summary of each class of pathogen, outlining the mechanisms of action for major classes of conventional antimicrobial drugs and the diverse evolutionary strategies which pathogens use to resist them. We conclude this part with a brief outline of alternative antimicrobial therapies, both established and emerging, and within this context, introduce pathogen targeting viruses as antimicrobial agents.

In the second part, we discuss the different types of pathogen-targeting virus—bacteriophages, virophages, mycoviruses, and parasite-associated viruses. We outline their mechanisms of action and present an up-to-date overview of the research and clinical landscape around their antimicrobial applications, with a particular focus on bacteriophages. Lastly, we discuss the pharmaceutical formulation considerations and clinical translation challenges associated with phage therapy, before clarifying the unmet needs and future directions in virus-based antimicrobial therapies.

## 2. Pathogens, Antimicrobials, and Antimicrobial Resistance

Human pathogens can be classified as cellular (bacteria, parasites, and fungi) or as acellular (predominantly viruses, but also prions) [10]. Bacteria, viruses, and parasites in particular are responsible for causing the most devastating infectious disease outbreaks, resulting in the cumulative deaths of billions of people, and have, therefore, had an immeasurable effect on the course of human history. Animal pathogens span the same basic classes, but (by definition) differ in the host organisms they affect, though there is considerable overlap in pathogens which may affect humans as well as animals. An estimated 60% of emerging infectious diseases are ‘zoonotic’ in nature [11], originating in animal species and later spreading to humans. Epidemics resulting from zoonoses are a regular and major health concern—the most notable recent example being the COVID-19 pandemic (caused by the severe acute respiratory syndrome coronavirus 2 or SARS-CoV-2 virus), which is believed to have originated in bats.

Prions are misfolded proteins, which can (upon contact) induce conformational changes in normal proteins of the same type. As there are no current treatments for prion diseases and as prions do not contain genetic information (and thus, cannot evolve to resist any hypothetical treatments), there are no associated issues with AMR and, therefore, prions will not be discussed further in this review.

Bacteria, fungi, viruses, and parasites, on the other hand, all contain genetic material which allows them to develop or acquire resistance to antimicrobial agents under the right circumstances. Transmission in humans is typically mediated by one or more of direct physical contact, contaminated objects/surfaces (fomites), via the respiratory pathway, by consuming contaminated food and water, through vectors such as insects, or from mother to child during pregnancy/birth. Pathogenic fungal infections are most commonly opportunistic in nature, and thus, predominantly infect immunocompromised individuals through environmental exposure, though some fungi can cause disease in immunocompetent hosts.

Pathogen transmission in animals is broadly similar to transmission in humans. Horizontal transmission is common and well-documented, with many bacterial and viral pathogens spreading through fomites or via the respiratory pathway. A significant proportion of infectious disease in animals is vector-borne, with ecto- (external) and endo- (internal) parasites responsible for transmitting a large variety of infectious diseases (e.g., tick-borne Babesia parasite or midge-borne Bluetongue virus) in both domestic and livestock animals. Faeco-oral transmission is another common pathway of infection, especially in food-producing animals where high-density housing and environmental exposure facilitates pathogen spread. Vertical transmission is also well-documented with numerous pathogens capable of crossing the placental barrier or being transmitted from mother to offspring through infected milk. Zoonotic transmission of pathogens from animals to humans may occur through direct contact, environmental exposure (e.g., consumption of contaminated animal products, via the respiratory pathway, handling of infected animals), as well as through vector transmission.

Antimicrobial agents exist for the prophylactic or curative use against infections by each of these classes of pathogens, and are aptly named antibacterials, antifungals, antiparasitics, and antivirals. Here, we will briefly outline each class of pathogen, the common antimicrobial agents used to combat them, and summarise the modes of resistance which pathogens have developed against them.

### 2.1. Bacteria

Bacteria are single-celled prokaryotic organisms, the majority of which do not require a host organism to replicate (extracellular bacteria). In contrast, intracellular bacteria can enter and replicate within host cells, and facultative bacteria can utilize both intra- and extracellular pathways. In the environment, bacteria are ubiquitous, and most environmental bacterial species are not pathogenic to humans. Bacteria may exist in a free-floating ‘planktonic’ state, or as part of a complex community called a ‘biofilm’. In simple terms, a biofilm is a protective assembly consisting of a population of bacteria embedded within a hydrogel-like matrix which contains a variety of components, such as polysaccharides, proteins, lipids, and extracellular DNA. Biofilms may offer protection to the resident bacterial colony from external stressors (including antibiotics), as well as facilitate the sharing of nutrients and horizontal gene transfer.

Bacteria which cause disease typically do so through the production of toxins, direct invasion and functional disruption of host tissues, and through the inadvertent damage inflicted by the hosts’ immune response. In terms of the global health threat posed by AMR, antibacterial resistance is of the highest concern due to a combination of factors, including the extensive large-scale use of antibiotics which drive the selection for resistance genes in medicine and industry, the efficiency of the resistance mechanisms evolved by bacteria, their typically rapid growth, as well as the facile nature of horizontal gene transfer between bacteria which allow resistance genes to proliferate. AMR in bacteria is an active global crisis.

Tuberculosis, which is a disease caused by *Mycobacterium tuberculosis* (*M. tuberculosis*), very likely has the highest cumulative death toll of all infectious diseases, estimated to be well over 1 billion. Cases of tuberculosis have been documented as far back as 8000 years ago, and it is still the cause of the highest number of deaths (ca. 1.23 million in 2024) [12] arising from infectious disease, particularly in less economically developed areas. Tuberculosis is extremely contagious and is easily spread through the air (inhaling bacteria expelled by an infected person, e.g., through coughing). It is increasingly challenging to treat due to the emergence of multi-drug resistant (MDR), extensively drug-resistant, or totally drug-resistant strains, which may require months or years of treatment with several concomitant antibiotics, or may have no effective treatment at all. This is particularly concerning, as up to a quarter of all people may already be infected with a latent form of tuberculosis, which may progress to active disease if the immune system becomes weakened (e.g., due to immunodeficiencies, receiving immunosuppressant medical treatment, or with advanced age) [13]. Rifampicin-resistant tuberculosis is a recent addition to the WHO bacterial priority pathogens list (2024).

Bovine tuberculosis (primarily caused by *Mycobacterium bovis*, and less commonly by other members of the *Mycobacterium tuberculosis* complex) is a zoonotic disease which spreads to humans either via inhalation of aerosols expelled by infected animals or through the ingestion of unpasteurised milk. Bovine tuberculosis is largely controlled in high-income countries through measures such as pasteurisation, surveillance, and culling programmes. However, it continues to pose a large healthcare and economic burden in low- and middle-income countries (LMICs), where it remains endemic across most of Africa and many parts of Asia. *M. bovis* is intrinsically resistant to pyrazinamide, one of the first-line treatment drugs for active TB), narrowing the treatment options available to infected humans. Bovine tuberculosis is listed by the World Organisation for Animal Health (WOAH) as a notifiable disease, reflecting its significance for animal health, trade, and global disease control efforts.

**Figure 2:**
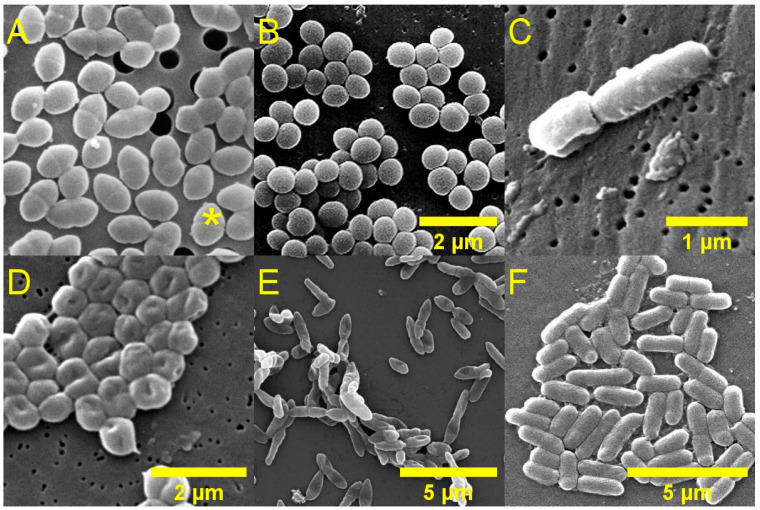
Electron microscopy images of selected ESKAPEE pathogens: (**A**). *E. faecium* (* scale unknown), (**B**). *S. Aureus*, (**C**). *K. pneumoniae*, (**D**). *A. baumannii*, (**E**). *P. aeruginosa* [17] (**F**). *E. coli.* Panels (**A**–**F**) adapted from public domain images from the Centers for Disease Control and Prevention Public Health Image Library (identification codes 209, 11154, 6834, 6498, and 8800, respectively). Image E adapted from Reference [17].

Organisations such as the United Kingdom Health Security Agency (UKHSA) [14] and the World Health Organization (WHO) [15] have published lists of resistant pathogenic bacterial species which are considered a public health priority for research and development. Pathogens which are rated as of high or critical importance are summarised in Table 1. A subset of these resistant species is responsible for the vast majority of hospital-acquired infections, and are referred to as the ESKAPEE pathogens, which is an acronym that stands for *Enterococcus faecium*, *Staphylococcus aureus*, *Klebsiella pneumoniae*, *Acinetobacter baumannii*, *Pseudomonas aeruginosa*, *Enterobacter* spp., *Escherichia coli* (Figure 2). *E. coli* is the cause of the largest estimated number of infections annually in England, according to the most recent ESPAUR report (2024–2025) [16].

*E. Coli* is a common commensal bacterium found in the gut of most mammals, although Shiga toxin-producing *E. coli* (STEC) can be pathogenic to humans. Enterohemorrhagic *E. coli* (EHEC), a subset of STEC, has higher virulence and are associated with especially severe disease in humans. The most prevalent EHEC serotype, O157:H7, is found commensally in the gut of sheep, cows, and other ruminants. Animals do not possess the necessary receptors for Shiga toxin to bind, so are asymptomatic when infected and, therefore, containment strategies are difficult to implement. In addition, STEC/EHEC are easily passed from animals to humans via direct contact or contaminated food produce, so farm animals present a very vast environmental reservoir of potentially pathogenic *E. coli*. Whole genome sequencing studies have identified STEC isolates carrying genes with phenotypic resistance to up to ten classes of antibiotic [18]. In the UK, recent surveillance data reported 533 confirmed cases of STEC 0157 in 2023 [19], and several food-associated outbreaks of other STEC serotypes [20,21].

**Table 1:** Selected pathogenic bacteria and their corresponding AMR concern rating (UKHSA, 2025) [14], priority level (WHO, 2024) [15], and class of antibiotic resistance (ESPAUR, 2025) [16].

Order	Family	Pathogen	AMR Concern (UKHSA Rating, 2025) [14]	Priority Level (WHO Rating, 2024) [15]	Resistance (ESPAUR Report 2025) [16]
*Enterobacterales*	*Enterobacteriaceae*	*Klebsiella pneumoniae*	Critical	Critical	Carbapenem
			-	Critical	3rd generation cephalosporins
		*Escherichia coli*	Critical	Critical	Carbapenem
			-	Critical	3rd generation cephalosporins
		*Salmonella enterica*	-	High	Fluoroquinolone
		*Non-typhoidal salmonella*	-	High	Fluoroquinolone
		*Shigella* spp.	-	High	Fluoroquinolone
		*Enterobacter* spp.	-	Critical	Carbapenem
			-	Critical	3rd generation cephalosporins
		*Citrobacter* spp.	-	Critical	3rd generation cephalosporins
	*Yersiniaceae*	*Serratia* spp.	-	Critical	3rd generation cephalosporins
	*Morganellaceae*	*Proteus* spp.	-	Critical	3rd generation cephalosporins
		*Morganella* spp.	-	Critical	3rd generation cephalosporins
*Pseudomonadales*	*Moraxellaceae*	*Acinetobacter baumannii*	Critical	Critical	Carbapenem
	*Pseudomonadaceae*	*Pseudomonas aeruginosa*	-	High	Carbapenem
*Neisseriales*	*Neisseriaceae*	*Neisseria gonorrhoeae*	High	High	Fluoroquinolone
*Bacillales*	*Staphylococcaceae*	*Staphylococcus aureus*	High	High	Methicillin
*Mycobacteriales*	*Mycobacteriaceae*	*Mycobacterium tuberculosis*	-	Critical	Rifampicin
*Lactobacillales*	*Enterococcaceae*	*Enterococcus faecium*	-	High	Vancomycin

#### 2.1.1. Antibiotics

Bacteria have a range of physical and metabolic features which can be selectively targeted by antibacterial agents. Unlike eukaryotic cells, most bacterial cells possess a cell wall, the structure of which can be used to classify bacteria into two broad categories. Some bacteria have a cell wall composed of a thick peptidoglycan layer, which readily retain Gram stain, and such bacteria are therefore known as Gram-positive. Gram-negative bacteria on the other hand, have a thin peptidoglycan layer surrounded by a lipid bilayer membrane, and do not readily uptake and retain the Gram stain. The outer lipid membrane of Gram-negative bacteria presents a physicochemical barrier to many classes of antibiotics, particularly large or hydrophilic molecules, contributing to their comparatively reduced susceptibility. Additionally, the outer membrane is composed in part of lipopolysaccharides (LPS), which are potent triggers of the immune system and contribute to the pathogenicity of Gram-negative bacteria such as *E. coli*.

Antibiotics may be broadly classified according to their mechanism of action, spectrum of activity, and clinical application, each of which has important implications for efficacy, toxicity, and the development of antibiotic resistance. Major classes of antibiotics and their modes of action are summarised in Table 2. The most widely used category of antibiotics interferes with the cell wall of bacteria by preventing the synthesis or crosslinking of constituent peptidoglycan molecules. The majority of these are the β-lactam antibiotics (which includes the penicillins, cephalosporins, and carbapenems) which typically have very low toxicity as they target peptidoglycan synthesis, which is absent in humans. The β-lactam component targets penicillin binding proteins in the cell walls of bacteria, which are variable in terms of their structure and affinity towards the β-lactam antibiotics.

Other commonly used types of antibiotics are those which can interfere with metabolic pathways (such as disruption of folate synthesis by sulfonamides), interfere with bacterial ribosomes to inhibit protein synthesis (e.g., tetracyclines, aminoglycosides, macrolides) and those which inhibit bacterial enzymes that are required for DNA or RNA synthesis (such as quinolones and fluoroquinolones). In some cases, off-target toxicity may occur due to overlap with human targets, which are evolutionarily related to their bacterial counterparts. For similar reasons, off-target toxicity issues are also associated with antibiotics that target and disrupt the bacterial cell membrane (such as polymyxins), as they rely on physicochemical interactions with the membrane that are not entirely bacteria-specific—consequently, these antibiotics are usually treated as last-resort.

Although antibiotics in current use predominantly utilise these modes of action, there are several novel targets currently under investigation. The LPS transport system in Gram-negative bacteria (which is made up of several proteins and is responsible for shuttling LPS components to the outer membrane) may be targeted to interfere with the formation of the outer membrane, thereby making Gram-negative bacteria more vulnerable. Several examples of LPS transport inhibitors which have reached phase 3 clinical trials exist, such as Murepavadin [22], which acts against *P. aeruginosa* (subsequently withdrawn due to nephrotoxicity concerns), and more recently Zosurabalpin [23] which is active against *A. baumannii*. Further targets, including the internal cell membrane [24] and energy production [25], are also currently being explored in clinical studies.

Further to their modes of action, antibiotics may be further classified by their spectrum of activity. Narrow-spectrum antibiotics exhibit activity against specific bacterial pathogen, without affecting the commensal microbiome—for instance, fidaxomicin which is used to treat *Clostridium difficile* infections. Conversely, broad-spectrum antibiotics such as doxycycline may have a much wider range of activity, spanning both Gram-negative and Gram-positive bacteria. From the perspective of antimicrobial stewardship, treatment of bacterial infections with narrow-spectrum antibiotics is preferable as it exerts a much lesser collateral selection pressure for AMR traits. In practice, however, this is not always possible, and broad-spectrum antibiotics are an invaluable resource for empirical therapy in cases where it is not possible or practical to use narrow-spectrum antibiotics (e.g., in severe infections where the infecting species are unknown).

Empirical antibiotic therapy is often utilised in veterinary practice, especially in cases where access to bacterial culture facilities is scant or not financially feasible. For instance, cefovecin is a broad-spectrum long-lasting antibiotic in the cephalosporin family, which offers clear compliance advantages in small animal practice due to its single-dose, injectable formulation; however, its widespread empirical use raises antimicrobial stewardship concerns [26,27]. As a third-generation cephalosporin, classed by WHO as a critically important antibiotic, this antibiotic has been associated with emerging resistance trends in companion animal samples. Two recent studies from Germany investigating cefovecin resistance in *E. coli* and *K. pneumoniae* isolated from veterinary samples found the proportion of resistance to be 11.6% and 16.3%, respectively, and up to 70% of *K. pneumoniae* isolated from feline urinary tract samples showed cefovecin resistance [28,29].

**Table 2:** Major antibiotic classes, their mechanisms of action, typical spectra of activity, and dominant antibacterial resistance mechanisms.

Antibiotic Class	Mechanism of Action	Typical Spectrum	Representative Examples	Dominant AMR Mechanisms	AMR Relevance/Notes
β-lactams	Inhibit cell wall peptidoglycan synthesis via penicillin-binding proteins	Broad (varies by subclass)	Penicillins, cephalosporins, carbapenems	β-lactamase production; altered PBPs; reduced porin permeability	Major drivers of clinical AMR; carbapenem resistance of critical concern
Glycopeptides	Bind peptidoglycan precursors, inhibiting cell wall synthesis	Gram-positive	Vancomycin	Target modification (e.g., altered D-Ala-D-Ala termini)	Resistance largely confined to Gram-positive pathogens
Aminoglycosides	Inhibit protein synthesis (30S ribosomal subunit)	Primarily Gram-negative; some Gram-positive	Gentamicin, amikacin	Enzymatic drug modification; reduced uptake; efflux	Resistance often plasmid-mediated
Macrolides	Inhibit protein synthesis (50S ribosomal subunit)	Gram-positive; atypical pathogens	Erythromycin, azithromycin	Ribosomal methylation; efflux pumps	Rapid resistance emergence in respiratory pathogens
Tetracyclines	Inhibit protein synthesis (30S ribosomal subunit)	Broad	Tetracycline, doxycycline	Efflux pumps; ribosomal protection proteins	Widespread resistance due to historic overuse
Fluoroquinolones	Inhibit DNA gyrase and topoisomerase IV	Broad	Ciprofloxacin, levofloxacin	Target site mutations; efflux; reduced permeability	Strong selection pressure; resistance increases rapidly
Sulfonamides	Inhibit folate biosynthesis	Broad	Trimethoprim–sulfamethoxazole	Enzyme mutation; alternative metabolic pathways	Resistance commonly linked to mobile genetic elements
Polymyxins	Disrupt bacterial outer membrane via LPS binding	Gram-negative	Colistin	LPS modification; membrane remodelling	Last-resort agents; resistance now emerging globally

#### 2.1.2. Antibiotic Resistance

There is an important distinction between intrinsic resistance which arises from the innate features of certain bacteria (and does not pose a threat in terms of AMR), and acquired resistance which emerges and spreads as a result of selection pressure exerted by exposure to antibiotics.

Intrinsic resistance accounts for the reduced natural susceptibility of some types of bacteria to particular antibiotics. For example, Gram-negative bacteria, by virtue of possessing a protective outer lipid membrane, have higher intrinsic resistance to the action of antibiotics as they are less able to permeate into the cell (this is particularly true of hydrophilic and large-molecule antibiotics). Likewise, β-lactam antibiotics that target cell wall synthesis are not effective against mycoplasma bacteria which do not possess a cell wall. Biofilm formation is another feature which is intrinsically protective against antibiotics—this occurs through several interconnected mechanisms, including poor drug penetration into the biofilm matrix and the survival of tolerant subpopulations which can re-establish the infection after treatment.

Acquired resistance characteristics, on the other hand, are problematic because their proliferation results in the gradual loss of antibiotic efficacy. The high bacterial cell density within biofilms coupled with the presence of extracellular DNA within the biofilm matrix helps to facilitate the emergence and proliferation of acquired resistance.

Broadly, as antibiotics must first bind to a bacterial target, one resistance strategy which has evolved is the mutation of targets in bacteria such that the affinity of the antibiotic to the target is reduced. For instance, vancomycin-resistance in *Enterococci* spp. may be mediated by a single amino acid substitution, which causes a 1000-fold decrease in vancomycin binding efficiency [30]. Between 1990 and 2021, the highest increase in deaths attributable directly to bacterial AMR was due to methicillin-resistant *S. aureus* (MRSA) [4], in which resistance is primarily conferred through changes to the *S. aureus* penicillin-binding protein.

Another widespread resistance mechanism is the production of enzymes which can inactivate antibiotics, for example β-lactamase enzymes that cleave the β-lactam structure in β-lactam antibiotics, as is the case for carbapenem-resistant *E. coli* [31]. Amongst Gram-negative bacteria, the highest increase in resistance between 1990 and 2021 was towards the carbapenem class [4]. It is also possible for bacteria to utilise naturally present efflux pumps (proteins in the cell membrane which transport substances out of the cell) to actively pump out antibiotic molecules [32] or modify naturally present porins (proteins in the outer membranes of Gram-negative bacteria that shuttle small, hydrophilic substrates) to reduce membrane permeability [33]. While detailed discussion of antibiotic resistance mechanisms in bacteria is beyond the scope of this review, they are addressed in detail elsewhere in the literature [34].

### 2.2. Fungi

**Figure 3:**
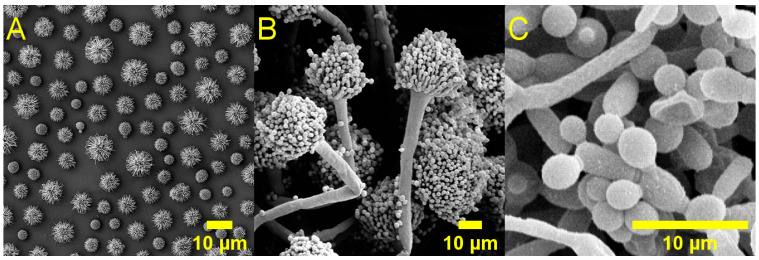
Electron microscopy images of (**A**). *Cryptococcus* spp. [35], (**B**). *Aspergillus fumigatus* (both under Creative Commons License 4.0) [36], and (**C**). *Candida albicans* [37]. Adapted with permission from relevant references.

Fungi are eukaryotic organisms which may be single-celled (yeasts, such as *Cryptococcus* spp.), multicellular (moulds, such as *Aspergillus fumigatus*), or able to adopt both yeast-like and mould-like morphologies (dimorphic fungi, such as *Candida albicans*); Figure 3. Fungi are independent organisms, and pathogenicity to animals is usually incidental rather than adaptive in nature. Most pathogenic fungi are opportunistic, only causing disease in patients with significant co-morbidities or compromised immune systems, such as those undergoing cancer treatment or suffering from acquired immunodeficiency syndrome. Typically, fungal diseases are caused by a combination of direct damage to host tissues as well as collateral damage from the host’s immune response. Fungi typically have slower growth rates than bacteria, are able to mechanically penetrate through tissues, and may also form biofilms. The latter generally results in infections that are more difficult to eradicate compared to bacterial infections.

Life-threatening fungal disease, such as invasive aspergillosis or candidiasis/candidemia (caused by members of the *Aspergillus* or *Candida* genera, respectively) is estimated to affect over 6.55 million people annually, directly accounting for 2.55 million deaths each year [6]. While there are many fungal species that affect immunocompetent and healthy individuals, it is very rare for such infections to lead to serious disease. Indeed, superficial fungal infections (such as skin infections or nail infections) are extremely prevalent in the general population (with more than 1 billion new infections yearly) [38], and the resultant economic burden of all fungal diseases is appropriately large, with an estimated yearly cost of upwards of 19 billion dollars in the United States alone [39].

Compared to bacteria, AMR in fungi may be considered as an emerging rather than active crisis, though the potential ramifications for public health are no less serious. There are relatively few available antifungal agents, and the emergence and proliferation of resistance, therefore, poses a significant threat to our continued ability to treat fungal disease. The most recent available WHO report (2022, Table 3) lists four critical priority fungal pathogens—*Cryptococcus neoformans*, *Aspergillus fumigatus*, *Candida auris*, and *Candida albicans*—of which, two in particular (*A. fumigatus* and *C. auris*) exhibit significant levels of antifungal resistance. This is alarming as the estimated annual incidences of invasive aspergillosis, candidiasis, and candidemia are very high (ca. 2.1 million, 939 thousand, and 626 thousand, respectively) [6].

*Batrachochytrium dendrobatidis* is a fungus which causes the disease chytridiomycosis in amphibians. It is particularly notable for having an uncharacteristically high degree of virulence at the population level, efficient waterborne transmission, as well as the ability to infect and cause severe and often lethal disease in immunocompetent hosts. Chytridiomycosis affects the skin of amphibians, which is vital for respiration and maintaining appropriate hydration and electrolyte balance. It is a highly prevalent disease, with an estimated 18.54% of the global population of amphibians affected [41] and is significant driver for declining amphibian populations and extinctions. Although *B. dendrobatidis* is largely restricted to amphibian hosts, its exceptional virulence and capacity to evade host immune defences illustrate the evolutionary potential of fungal pathogens, underscoring the broader risk of emerging fungal diseases.

Most clinically important invasive fungal pathogens are not true zoonoses but environmentally acquired infections with animal-associated reservoirs. Fungi such as *Histoplasma*, *Cryptococcus*, and *Aspergillus* spp. persist in organic matter that may be enriched by animal activity or faeces, and outbreaks of *Cryptococcus* spp. infection have been documented in both domestic and companion animals (most commonly cats) [42]. Human infection occurs predominantly via inhalation rather than direct animal-to-human transmission; nonetheless, animals play important roles as ecological reservoirs and amplifiers, underscoring the relevance of fungal diseases within a One Health framework.

#### 2.2.1. Antifungals

As eukaryotic organisms, fungal cells share many features with animal cells, which limits the available targets that can be exploited for effective antifungal therapy and presents higher potential for toxicity to host cells. For this reason, many antifungal drugs exhibit side effects associated with toxicity to host cells. Major classes of antifungal agents are shown in Table 4.

Among the most selective antifungal agents are the echinocandins, which inhibit the production of β-glucan in the fungal cell wall, in a manner analogous to β-lactam antibiotics. Another class of antifungal drugs is the polyenes (most notably amphotericin B) which bind to ergosterol in the fungal cell membrane and disrupt the integrity of the membrane with respect to metabolite and ion movement. This class of antifungal drugs also binds to the human equivalent cell membrane component (cholesterol), which results in relatively high toxicity and, therefore, limits the dose that may be administered. Recently, a new antifungal agent in the polyene class (mandimycin) [43] was discovered. Mandimycin targets various phospholipids in the fungal cell membrane rather than ergosterol, exhibiting higher selectivity towards the fungal cell membrane in vivo and, therefore, leading to significantly lower toxicity than amphotericin B.

Azoles (e.g., fluoconazole) and allylamines (such as terbinafine) both inhibit enzymes in the ergosterol biosynthesis pathway (P450 and squalene epoxidase, respectively), and both classes of antifungals may exert a degree of toxicity through interacting with the human equivalent enzymes in the cholesterol biosynthesis pathway. Drugs in these classes account for the vast majority of clinically used antifungal agents, though others exist and have a variety of mechanisms of action, some of which are not well understood. An as-yet experimental class of antifungals called the orotomides acts by inhibiting the growth of hyphae (the root-like structures formed by most fungal species). The first of this class of antifungals to be developed, olorofim, is currently in phase 3 clinical trials, with promising results from prior studies indicating good antifungal efficacy toward *Aspergillus* spp., including in patients with azole-resistant strains [44]. However, due to differences in the target enzyme, orotomides do not exhibit activity against *Candida* and *Cryptococcus* species. A recently reported class of antifungal agents, the coniotins (which work by targeting β-glucan in the fungal cell wall), appear to exhibit broad-spectrum activity against multiple critically important fungal pathogens, including *C. albicans*, *C. neoformans*, MDR *C. auris*, and *A. fumigatus* [45].

#### 2.2.2. Antifungal Resistance

As with bacteria, some fungal species exhibit naturally lower susceptibility to antifungals through intrinsic resistance mechanisms, such as reduced access/permeability which blocks antifungals from reaching their cellular target. In *Cryptococcus*, intrinsic resistance toward echinocandins is not yet fully understood, but it is thought to be related to the increased structural integrity of their cell walls and membranes [46]. Biofilm-forming fungal species also show high degrees of intrinsic resistance, in part due to reduced access but also due to several other contributing mechanisms which occur within the complex biofilm environment, for example in *Candida* [47]. Other intrinsic resistance mechanisms include the presence of altered target proteins (thus reducing binding ability) and cell membranes which have a naturally lower ergosterol content (hence less susceptibility towards azoles and polyenes). Susceptibility to antifungal agents may be reversibly lowered through adaptive stress-response mechanisms in fungi, which may result in treatment failure and, therefore, increase the chances of AMR trait emergence. There are several mechanisms which may contribute to such antifungal tolerance, described in detail elsewhere (for instance in *A. fumigatus* [48] and *C. albicans* [49]).

Acquired resistance mechanisms in fungi are generally analogous to those found in bacteria, and are most prevalent and clinically impactful in relation to azole drugs. For example, AMR may arise from modification of cellular targets such as the sterol 14α-demethylase enzymes, the target of antifungals in the azole class. Genetic changes to the sequences which encode these isozymes and thereby lead to azole resistance have been reported extensively in C. albicans, A. fumigatus, as well as in C. auris, and C. neoformans [50]. Overexpression of the target is also associated with resistance [51], providing capacity to maintain essential processes and requiring higher doses of antifungals for inhibition. Analogously, echinocandin resistance in *Candida*, *Cryptococcus*, and *Aspergillus* spp. may be mediated by changes to a subunit of the 1,3-β-glucan synthase enzyme [50]. Antifungal resistance can additionally be facilitated by the upregulation of various efflux pumps (particularly for azole drugs) [52], which serve to remove antifungal agents from the intracellular space and thereby reduce their effectiveness. Compared with bacteria, the spread of resistance traits in fungi is typically slower and occurs primarily through clonal expansion rather than horizontal gene transfer.

### 2.3. Viruses

Viruses are the most rudimentary pathogens in terms of their composition, lacking cellular structure and the ability to proliferate outside of host cells. Despite this, viruses have an extraordinary degree of genetic diversity, and can infect all known life forms, from single-celled organisms such as bacteria, to complex, multicellular organisms including plants and animals. Of all biological entities on Earth, viruses are by far the most abundant, estimated at approximately 10^31^ individual particles [53], an order of magnitude higher than the estimated number of all living cells (ca. 10^30^) [54]. It is unsurprising then that the global virome hosts a vast range of genetic diversity—giving rise to some 14,690 currently recognised species (at time of writing) [55], and very likely more than 10^6^ projected species (according to current knowledge) [56].

Many viruses exist as free infectious particles, and eukaryotic viruses typically enter host cells whole and subsequently disassemble. Once inside the cell, the genetic material of the virus is used to instruct the host cell to produce components necessary for the replication of the virus. In many cases, this eventually results in the release of a large cohort of virus progeny, either through the rupture of the host cell (lysis) or other mechanisms such as exocytosis. As viruses reproduce intracellularly using a host cell’s innate processes, it is difficult to selectively target pathogenic viruses without damaging the host organism. Broadly, viruses may be classified according to their genome type and replication strategy: RNA viruses, DNA viruses, and reverse-transcribing viruses. Reverse-transcribing viruses may have either RNA or DNA genomes, but are distinguished by their replication mechanism, which involves reverse transcription of their genome, sometimes followed by integration into the host genome (in retroviruses such as HIV).

**Figure 4:**
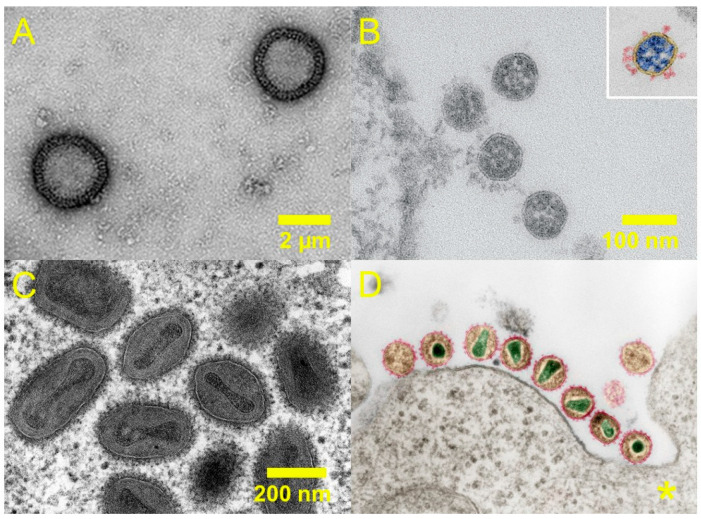
Electron microscopy images of selected viruses: (**A**) H1N1 influenza virus [59], (**B**) SARS-CoV-2 virions [60], (**C**) Variola major virus [61], (**D**) HIV-1 (* scale unknown) [62]. Panels (**A**) and (**B**) adapted from references [59,60], respectively (open access). Panels (**C**,**D**) adapted from electron microscopy images by the Robert Koch Institute (references [61,62]).

The pathogenic effect of viruses on humans and animals can be a result of a combination of direct tissue damage, injury from the immune response to the viral infection, as well as the loss of function of critical cell types, among other mechanisms. The Great Influenza Pandemic (1918–1920), killed up to 50 million people by some estimates [57], and was caused by a strain of the influenza A (subtype H1N1) virus. It is thought to have elicited an extreme immune response which subsequently damaged the lung tissue, rendering patients unable to breathe and vulnerable to opportunistic infections. Influenza A is also zoonotic, with large avian and swine reservoirs from which novel strains periodically emerge and infect humans [58]. Selected pathogenic viruses are shown in Figure 4, and selected pandemic/epidemic potential and pathogen priority ratings are summarised in Table 5.

Another notable example of a now eradicated viral disease is smallpox, caused by the Variola virus, which resulted in the deaths of hundreds of millions of humans over the course of history primarily through the effects of viral cell lysis, and severe disfigurement in those who survived. A vaccine against smallpox was the first vaccine to ever be developed, and to date, smallpox is the only infectious human disease that has been globally eradicated, a feat achieved through concerted vaccination and containment initiatives.

Antiviral resistance is less pervasive than antibiotic or antifungal resistance, in part due to the much more restricted use of antivirals than antibiotics/antifungals. It is primarily problematic in the context of chronic viral infection management, driven by exposure of viruses to long-term treatment and potentially incomplete suppression, for instance in HIV and viral hepatitis (hepatitis B and C). With the advent of combination antiretroviral therapy for HIV treatment, resistance has become relatively rare, but in certain contexts (such as with poor treatment adherence or with prior treatment failure) resistance to dolutegravir (a first-line antiretroviral drug) is rising. A recent analysis of the literature pertaining to dolutegravir resistance rates in LMICs has found that resistance trait emergence is less than 1% at the population level [63], and that continued dolutegravir resistance may be effectively mitigated against by measures at the individual level (e.g., support in treatment adherence).

**Table 5:** Selected pathogenic viruses and their respective pandemic/epidemic potentials (UKHSA, 2025) [14] and pathogen priority ratings (WHO, 2024) [64]. * Variola virus has been eradicated, but is considered high-risk if release from storage occurs.

Order	Family	Pathogen	Pandemic/Epidemic Potential (UKHSA Rating, 2025) [14]	Priority Pathogens: Public Health Emergency of International Concern Risk (WHO Rating, 2024) [64]
*Rowavirales*	*Adenoviridae*	Human Adenovirus (B, C, E, F)	Medium/High	-
*Hareavirales*	*Arenaviridae*	Mammarenavirus lassaense	-	High
	*Nairoviridae*	CCHF virus	-	High
	*Phenuiviridae*	SFTS virus	-	High
*Picornavirales*	*Caliciviridae*	Norovirus	Medium/High	-
	*Picornaviridae*	Enterovirus D68, A71	High/High	Not priority; Medium
*Nidovirales*	*Coronaviridae*	MERS-CoV	High/High	High
		SARS-CoV	-	High
*Mononegavirales*	*Filoviridae*	Ebola virus	Low/High	High
		Sudan ebolavirus	-	High
		Marburg virus	-	High
		Ravn virus	-	High
	*Paramyxoviridae*	Nipah virus	High/High	High
	*Pneumoviridae*	Human metapneumovirus	Medium/High	Not priority; Low-Medium
*Amarillovirales*	*Flaviviridae*	Dengue virus	Low/High	High
		Zika virus	Low/High	High
		Yellow fever virus	-	High
		Hepatitis C virus	Low/High	-
*Articulavirales*	*Orthomyxoviridae*	Non-seasonal infuenza	High/High	High
*Chitovirales*	*Poxviridae*	Monkeypox virus (Clade 1)	Medium/High	High
		Variola virus	-	High *
*Elliovirales*	*Hantaviridae*	Sin Nombre virus	-	High
		Hantaan virus	-	High
*Martellivirales*	*Togaviridae*	Chikungunya virus	-	High
		VEE virus	-	High

#### 2.3.1. Antivirals

The lifecycle of eukaryotic viruses may be broadly summarised as follows. A free virus particle must first attach itself to specific receptors on the host cell surface, and subsequently enter the cell (typically through endocytosis or membrane fusion). Once inside the cell, the virus may undergo ‘uncoating’ or disassembly to release its genome, which is then expressed and replicated. Newly synthesised viral genomes and components are assembled into progeny virions, after which they undergo a series of maturation processes and are then released from the host cell by lysis or by mechanisms such as exocytosis.

In general, antiviral drugs tend to have a relatively narrow spectrum of activity, and may target different stages of the viral lifecycle, for example by interfering with the attachment and entry processes. The attachment inhibitor drug fostemsavir (typically given to patients with MDR HIV) binds to a capsid protein subunit (gp120) on the surface of the HIV type 1 virus (HIV-1), preventing it from binding to the CD4 host cell receptor [65]. Similarly, antiviral drugs may inhibit the entry of viruses into host cells by binding to proteins which are needed to facilitate fusion or entry (e.g., enfuvirtide). Antiviral drugs which act on these stages of the lifecycle are relatively limited in scope.

Many common antiviral agents act by inhibiting processes or enzymes to disrupt viral genome replication. For example, nucleotide/nucleoside analogues (the most used class of antivirals) target polymerase enzymes to become incorporated into new RNA/DNA strands in place of native nucleotides. This causes downstream effects, such as the premature termination of the growing RNA/DNA strand or inhibition of the polymerase enzyme; therefore, preventing viral genome replication. There are some concerns around the toxicity of nucleoside/nucleotide analogues, primarily due to potential inhibition of human mitochondrial polymerase [66], though this is largely drug dependent, as in some cases viral kinase enzymes are required to activate the drug thereby restricting effects to infected cells only (e.g., acyclovir).

In contrast, non-nucleoside polymerases or reverse-transcriptase inhibitors act via allosteric binding to viral enzymes and typically exhibit a narrower, virus-specific spectrum of activity, with resistance arising rapidly through point mutations at the inhibitor site. Some antiviral agents act earlier in the viral lifecycle by targeting capsid stability or uncoating; however, such drugs have largely fallen out of use due to the rapid emergence of resistance, often mediated by single amino acid substitutions in capsid proteins, as exemplified by amantadine resistance circulating in influenza A strains.

Protease inhibitors are drugs which are typically used in the management of chronic viral infections such as HIV and hepatitis C, and work by inhibiting protease enzymes from converting viral protein precursors into functional viral proteins during maturation. Integrase inhibitors (such as dolutegravir) are used for the management of HIV, and work by preventing the integration of viral DNA into the host genome (which is required for the virus to replicate).

The major classes of antiviral drugs and their properties are summarised in Table 6.

#### 2.3.2. Antiviral Resistance

Compared to bacteria and fungi, the mutation rates in viruses are very high. This is particularly true of many RNA viruses and retroviruses that have polymerase and reverse-transcriptase enzymes which are incapable of proofreading and correcting replication mistakes [67]. Consequently, resistance traits may emerge and be selected for very rapidly at the patient level—particularly in response to therapy with a single antiviral agent (monotherapy).

However, in contrast to bacteria, the proliferation of resistance traits in viruses is almost exclusively vertical, as horizontal gene transfer between viruses is rare. The spread of antiviral resistance traits at the population level, therefore, is often slower than in bacterial pathogens, and is constrained by the narrow ecological niche and host specificity of most viruses. For this reason, the emergence and proliferation of resistance traits in viruses can usually be effectively curtailed through treatment approaches that present a higher resistance barrier (such as using combination therapy), and through improvements in formulation and dosing which in turn improve patient compliance [68].

Intrinsic resistance features in some viruses include the lack of viral kinases (thus resistance to certain nucleoside analogue polymerase inhibitors), as well as polymerases with enhanced proofreading abilities (which reduce the incorporation of nucleotide analogues during replication). Some viruses are naturally less vulnerable to non-nucleoside polymerase or reverse-transcriptase inhibitors, because they lack an appropriate binding site in their polymerase. For instance, non-nucleoside reverse transcriptase inhibitors such as etravirine are effective against HIV-1 but not HIV-2 (two types of HIV with differences including surface antigens), as the binding site in the latter is too constricted spatially to allow drugs to bind [69]. Viruses which do not require proteolytic maturation or do not integrate their genome into the host DNA will not be affected by protease and integrase inhibitors, respectively.

The repertoire of resistance mechanisms accessible to viruses is more limited than those available to bacteria and fungi, as viruses encode relatively few proteins and have vastly smaller genomes. Acquired resistance in viruses is, therefore, predominantly mediated by modification of viral proteins, such as drug targets, like capsid proteins or enzymes, and to a lesser extent by other mechanisms such as changes to polymerase function, loss of kinase enzymes, or the emergence of compensatory mutations that can offset the fitness cost of resistance traits.

### 2.4. Parasites

**Figure 5:**
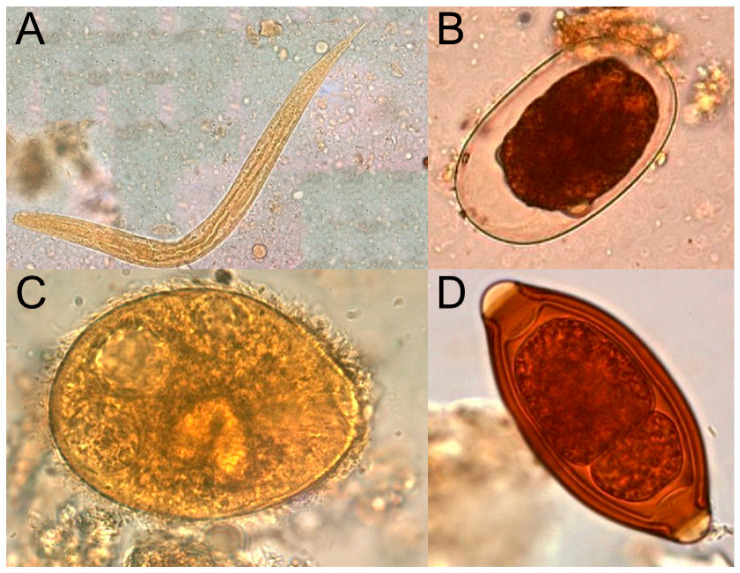
Micrographs of various parasites: (**A**) Strongyloides stercoralis (threadworm), (**B**) Hookworm egg, (**C**) Balantidium coli, and (**D**) Trichuris trichiura (Whipworm). Adapted from public domain images from the Oregon State Public Health Laboratory (reference [71]).

Parasites are a diverse range of eukaryotic organisms which lead a parasitic lifestyle—deriving benefit, such as nutrition, from a host organism at the host’s expense. They may be subdivided into endo- (internal) and ecto- (external) parasites, which live within or on the surface of a host organism, respectively. Further, they may also be categorised as single-celled protozoan parasites or multicellular animals such as lice, fleas, and helminths (worms, for instance tapeworms or pinworms). Micrographs of selected parasites are shown in Figure 5.

Some parasites, namely ectoparasites such as fleas or ticks, can also act as vectors for other pathogenic diseases thereby contributing to the dissemination of infections caused by antimicrobial-resistant pathogens. As ectoparasitic infections are typically superficial and do not usually cause severe disease directly, they tend to occupy a more peripheral position in discussions relating to antiparasitic resistance (and AMR more generally). However, the extensive use of ectoparasiticides in both veterinary and human medicine has generated a strong selective pressure and rapid emergence of resistance traits, which continue to undermine vector control efforts and threaten agricultural and aquacultural yields [70].

Compared to bacteria, fungi, and viruses, the life cycles of many parasites can be complex, often involving several distinct life stages that may take place within environmental reservoirs, within vectors, and ultimately within the host organism. Reflecting this, the transmission routes of parasites are also diverse. Many parasitic infections are transmitted through blood-feeding vectors such as mosquitoes, fleas, and ticks. As such, the incidence of diseases caused by vector-transmitted parasites is typically restricted by the ecological niche of the vector, which in turn is sensitive to environmental changes—something which is particularly prevalent in the modern era. Other transmission routes include but are not limited to foodborne, faeco-oral, zoonotic, mother-to-child, or host-to-host transmission via close personal contact.

In humans, the majority of severe disease and death arising from parasitic infection is typically caused by protozoans, with the highest burden arising from *Plasmodium* spp. the causative organisms behind malaria. Malaria is transmitted to humans by mosquitoes, with the parasite initially infecting and proliferating within liver cells. From there, the parasite progeny infects and damages the red blood cells of the host, causing various pathological effects, and potentially organ failure and death (especially from infections by *Plasmodium falciparum*). Incidence rates of malaria globally have been rising recently, with an estimated 282 million infections in 2024, representing an increase of around 9 million cases from the previous year [72]. The prevalence of malaria is highest in sub-Saharan Africa, accounting for some 95% of all cases, and it is also endemic in many countries in South Asia and Central/South America. Concerningly, resistance is now common to most classes of antimalarial drugs, and partial resistance is now emerging towards the current first-line treatment of artemisinin combination therapies, underscoring the fragility of existing treatment options against AMR. Other examples of protozoal diseases include leishmaniasis and giardiasis, with both organisms having developed strains which are resistant to first-line treatments (antimonials and metronidazole/albendazole, respectively) [73,74].

Another class of parasites with rising rates of antiparasitic resistance, especially in veterinary medicine but increasingly also in humans, are helminths. Antihelminthic resistance poses a large threat to the livestock industry, with helminth infections capable of causing severe disease (e.g., lungworm in cattle) and consequently vastly reduced productivity. Additionally, companion animals (such as cats and dogs) may host several zoonotic parasitic infections with identified antiparasitic resistance traits, such as hookworm (*Ancylostoma caninum*), heartworm (*Dirofilaria immitis*), and flea tapeworm (*Dipylidium caninum*) [75].

#### 2.4.1. Antiparasitics

As with antifungals, the eukaryotic nature of parasites makes them more difficult to selectively target than bacteria due to substantial biological and genetic overlap between parasite and host targets. There are relatively few antiparasitic drugs available, many of which have a narrow therapeutic spectrum. Consequently, the emergence of antiparasitic resistance has the potential to rapidly deplete available treatment options, with limited therapeutic alternatives.

Due to the stark biological differences between ectoparasites, helminths, and protozoa, it is common to divide antiparasitics based on their target organism in the first instance, followed by their mechanisms of action. Antiparasitics may thus be broadly subdivided into antiprotozoals and antihelminthics (for the treatment of endoparasites), and ectoparasiticides.

Antiprotozoal drugs are typically highly specific to the pathogenic protozoan, limiting cross-coverage between different protozoan infections. In malaria, *Plasmodium* parasites invade red blood cells and digest host haemoglobin, releasing free heme in the process. Artemisinin drugs work by binding to the heme and triggering the release of free radical species which directly damage and kill the parasite. In contrast, quinoline antimalarials (such as chloroquine and quinine) interfere with heme detoxification by preventing its crystallisation into inert hemozoin. The resulting build-up of free heme within the parasite food vacuole is lethal to the parasite at high concentrations [76].

Nitroimidazoles (e.g., metronidazole), on the other hand, have a relatively broad spectrum of activity compared to most antiprotozoals. They are used commonly in the treatment of protozoal infections such as giardiasis, amoebiasis, and trichomoniasis (caused by *Giardia duodenalis*, *Entamoeba histolytica*, and *Trichomonas vaginalis*, respectively). They act as prodrugs which are activated inside the parasites via redox enzymes, producing reactive intermediates which damage DNA and disrupt essential cellular processes [76].

Pentavalent antimonial drugs were historically the first-line drugs for the treatment of leishmaniasis (*Leishmania* spp.), though resistance is emerging rapidly in some parts of the world (especially within the Indian sub-continent, with treatment failure rates as high as 65% in some regions) [77]. The mechanism of action of these drugs is not fully elucidated, but is thought to involve the inhibition of trypanothione reductase enzymes, which are essential for the detoxification and oxidative stress defence processes in *Leishmania* [78].

Antihelminthic drugs typically display a broader spectrum of activity than antiprotozoals, and are often effective against protozoal species, too. The benzimidazoles (e.g., mebendazole) act primarily by binding to β-tubulin proteins and, therefore, inhibiting their polymerisation into microtubules, which are essential to facilitating processes such as glucose uptake, and maintaining cellular homeostasis [79]. Macrocyclic lactones, such as ivermectin, act on glutamate-gated chloride channels, causing the influx of chloride ions into cells and subsequent membrane hyperpolarisation and eventual death of parasites [80]. Both classes of antihelminthic exhibit activity across multiple helminth species, and are commonly employed as whole-flock prophylactic treatments to control gastrointestinal helminths in livestock animals. As a result, resistance to both ivermectin and benzimidazoles in livestock populations around the world is rising [81,82,83].

Neonicotinoids are widely used in veterinary medicine to control fleas and ticks, and act as agonists of nicotinic acetylcholine receptors in the nervous systems of insects. This causes persistent receptor activation and leads to neuronal depolarisation and eventual paralysis and death. Pyrethroids similarly target the insect nervous system by prolonging the opening of voltage-gated sodium channels, resulting in persistent neuronal firing and excitation. In contrast, phenylpyrazoles (e.g., fipronil) and isoxazolines act by inhibiting (γ-aminobutyric acid)-gated chloride channels (as well as glutamate-gated chloride channels in the case of isoxazolines), disrupting inhibitory neurotransmission and causing hyperexcitation, paralysis, and death. In the UK, *Ctenophalides felis* is the most common flea, and affects both canines and felines. Fipronil is widely available without prescription, and has high treatment failure rates which may be associated with resistance [84] and poor compliance/misuse [85].

The major classes of antiparasitic drugs and their associated properties are summarised in Table 7.

#### 2.4.2. Antiparasitic Resistance

Relative to bacteria, viruses, and—to a lesser extent—fungi, the emergence and spread of antiparasitic resistance in parasites is generally slower, reflecting their higher generation length and more complex life cycles. However, due to the very limited redundancy in available antiparasitic treatments, and the potential of antiparasitic resistance to severely impact agricultural productivity, it is still an issue of great concern.

In protozoans, antiparasitic resistance is perhaps best documented in *Plasmodium falciparum.* The development of resistance to first-line antimalarial treatments has unfortunately occurred on multiple occasions. Mutations which increase the drug efflux from the parasite digestive vacuole—thereby preventing inhibition of haeme detoxification—are responsible for chloroquine [86] and mefloquine resistance [87]. Artemisinin resistance has now also emerged and is primarily associated with mutations in genes encoding the Kelch13 protein, although the resulting resistance phenotype appears to arise from multiple, incompletely understood, mechanisms [88]. Many mutations are known for *Leishmania* spp. which are associated with resistance against a variety of drugs [89], for example resistance against antimonial drugs via increased efflux pump expression, decreased uptake by downregulation of aquaglyceroporin 1, and upregulation of antioxidant enzymes to deactivate reactive oxygen species. Resistance to nitroimidazole drugs is often mediated by either the loss or impairment of enzymes required for drug activation, or by increased efflux to remove the drugs from the intracellular space (e.g., in *Giardia duodenalis* [73], *Trichomonas vaginalis* [90,91], and *Entamoeba histolytica*) [92].

The primary and most widely reported mechanism conferring resistance to benzimidazoles in helminths involves mutations that modify the β-tubulin target, therefore, reducing the binding ability of benzimidazole drugs, which has been reported for a variety of helminth species across the world [79]; for instance, in canine hookworm in the United States of America [93]. Fenbendazole resistant pinworms are a particularly well-known problem in horses [94,95]. Resistance towards macrocyclic lactones may be mediated by mutations which upregulate the expression of efflux pumps [96], or which alter the target site (glutamate-gated channels) to reduce drug binding efficiency [97], among other mechanisms.

While the efficacy of neonicotinoids remains high, resistance in insect populations is typically mediated by increased enzymatic detoxification [98]. For instance, a study on sheep blowfly has linked resistance toward imidacloprid (a neonicotinoid) to increased expression of cytochrome P450-encoding genes, which are implicated in metabolic detoxification of the drug (and consequently a reduction in efficacy) [99]. Similarly, enhanced enzymatic detoxification has been implicated in cases of pyrethroid resistance (e.g., permethrin resistance in ticks) [100], in addition to mutations which alter the sodium channel target [101].

### 2.5. Alternative Antimicrobial Therapies

In addition to conventional antimicrobial drugs, a variety of other therapies are available that may reduce the reliance on antimicrobials. Selected approaches, both established and emerging, are briefly summarised here.

#### 2.5.1. Established

Vaccines, which stimulate the host immune system using weakened, inactivated, or molecular components of microbes, are a widely used and established strategy to prevent disease or reduce infection severity, thereby indirectly reducing antimicrobial consumption and the associated selection pressure. Vaccines are available against a variety of pathogens, including viral (e.g., influenza, SARS-CoV-2), bacterial (e.g., *M. tuberculosis*, *S. pneumoniae*), and parasitic (e.g., *P. falciparum*), in both human and animal medicine. However, many pathogens are not amenable to vaccine development—for instance, those which have high antigen variability such as HIV, or pathogens unable to elicit long-term immunity in a host. Vaccines against fungal infections are particularly challenging to develop, and thus far are limited mostly to pre-clinical applications [102].

Antibodies are proteins produced by the immune system which are highly specific for particular ‘antigens’, which in the context of infectious disease can include proteins, polysaccharides, or toxins expressed by a pathogen. When antibodies bind to specific antigens on a pathogen, they may block pathogen attachment or entry into host cells, neutralise toxins, and act as tags to facilitate the removal of the pathogen by phagocytic immune cells. Monoclonal antibodies are, essentially, large quantities of a specific antibody which are produced for therapeutic purposes. Unlike antimicrobials, monoclonal antibodies have minimal impact on the commensal microbiome and limited off-target toxicity. However, production is resource-intensive and costly, and typically administration is intravenous, preventing widespread use. In addition to applications outside of infectious disease, their use is already established in the clinic for the treatment of selected viral and bacterial infections such as sotrovimab for SARS-CoV-2, and bezlotoxumab for recurrent *C. difficile*.

Faecal microbiota transplantation, or the transfer of healthy donor stool material into the intestines of a patient, is a highly effective strategy used to treat recurrent *C. difficile* infections [103,104]. It is being increasingly explored for the treatment of other gastrointestinal infections [105,106] where microbiota imbalance may complicate treatment or increase disease severity.

Adjunct therapy (sometimes called adjuvant therapy) is administered alongside a primary therapy and is intended to modulate the immune response of the host to improve therapeutic outcomes. For example, steroids may be given to patients with severe infections to alleviate inflammation caused by the immune response, which may contribute to mortality [107]. Conversely, immunostimulatory agents which support phagocyte function may be given to aid pathogen clearance [108,109].

Antimicrobial peptides are a broad class of naturally occurring proteins which are part of the defence arsenal against infectious diseases in many organisms, including animals, bacteria, and fungi. In humans, they have complex mechanisms of action, involving both immunomodulatory and direct antimicrobial action aspects. Antimicrobial peptides may be natural (isolated from organisms) or synthetic. Although their clinical use remains limited, several antimicrobial peptides are already approved, including polymyxins for the treatment of multidrug-resistant bacterial infections and enfuvirtide as an antiviral entry inhibitor.

#### 2.5.2. Emerging

Novel drug delivery and formulation approaches may be exploited to deliver conventional antimicrobials in a manner which increases their efficacy and exerts less selective pressure for AMR traits. For instance, using carrier nanoparticles which may have targeting moieties to selectively deliver drugs to pathogens [110], or better biofilm penetrating abilities than free drug [111]. Alternatively, stimuli-responsive systems such as those which release drug in response to changes in pH in the infection microenvironment [112] or the presence of bacterial enzymes have been used to substantial effect [113]. Smart delivery systems such as these can for instance restrict the exposure of commensal microbes to antimicrobials, thereby decreasing the risk of AMR trait emergence/proliferation.

The primary aim of antivirulence therapy, as opposed to conventional antimicrobial therapy, is to decrease the virulence of pathogens, rather than to eliminate them. One example of antivirulence agents is quorum sensing inhibitors, which disrupt the cell-to-cell communication in bacteria (quorum sensing) that is an important mechanism used by bacterial colonies to modulate their gene expression as a unit. Quorum sensing inhibitors can therefore downregulate the expression of a variety of virulence factors in a bacterial population, such as the formation of biofilm [114] or antibiotic tolerance [115].

CRISPR-Cas technology (“clustered regularly interspaced short palindromic repeats”) is an established gene-editing method, derived from natural adaptive immune defence mechanisms of microorganisms, which is now being explored as an antimicrobial therapeutic strategy. Briefly, CRISPR-Cas utilises guide RNA sequences to direct Cas enzymes to specific nucleic acid targets, where they selectively damage parts of the pathogen DNA/RNA. Several CRISPR-Cas systems have entered early translational development, for example to treat HIV [116] or for the treatment of urinary tract infections by *E. coli* [117]. More broadly, the field of RNA-based therapeutic platforms as direct antimicrobials is still relatively nascent, with progress to date mostly limited to experimental approaches targeting viral infections.

In veterinary medicine, nematophagous fungi (fungi which feed on nematodes or their eggs) have been successfully used as an alternative to conventional antihelminthics to control infestations in a variety of livestock [118,119,120,121]. Biological control methods may also be vector-directed, for example the use of parasitic *Wolbachia* bacteria to infect mosquitoes, which reduces their ability to act as vectors for human pathogens such as Dengue virus [122].

Among emerging strategies for infection treatment and control, viruses that naturally infect and regulate microbial populations are increasingly regarded as among the most viable alternatives to conventional antimicrobials. Such viruses, by virtue of being biological entities themselves, offer a fundamentally different approach to combating AMR—as they can evolve alongside pathogens, and thus may overcome resistance mechanisms that limit the long-term efficacy of conventional antimicrobials. Bacteriophages (viruses which infect bacteria) have a long history of use in both human and animal medicine, and are currently employed in the clinic in compassionate-use cases to treat severe and MDR infections. Moreover, phages offer numerous advantages over other types of therapy—including their high pathogen specificity, self-amplifying nature at the site of infection, and relatively low production costs. The second part of this review considers the therapeutic applications and formulation strategies of pathogen-targeting viruses.

#### 2.5.3. Transitioning to Virus-Based Therapies

Despite the breadth of available alternative strategies for antimicrobial therapy, most still rely on mechanisms which are vulnerable to the same evolutionary pressures which drive AMR, potentially constraining their long-term efficacy. In contrast, viruses that naturally infect pathogenic microbes represent a fundamentally distinct therapeutic paradigm: they are capable of co-evolving with their targets and are often highly selective. Among these, bacteriophages have shown the greatest translational promise and clinical maturity, but virophages, mycoviruses, and parasite-associated viruses are increasingly recognised for their potential to modulate or suppress infections across other pathogen classes.

## 3. Viruses for Antimicrobial Therapy: Bacteriophages, Virophages, Mycoviruses, and Parasite-Associated Viruses

**Figure 6:**
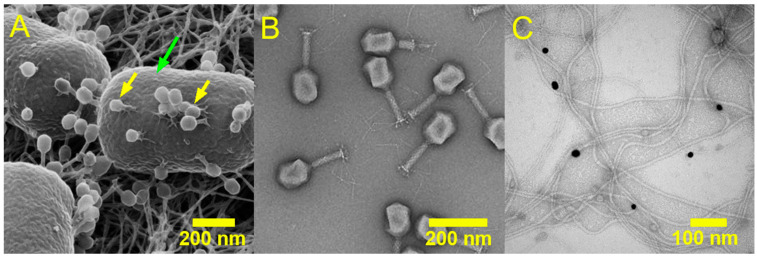
(**A**) Helium-ion microscopy image of *E. coli* (green arrow) with T4 phages (yellow arrows) [123], (**B**) Enterobacter cloacae phages [124], and (**C**) filamentous M13 phages (active against *E. coli*) interacting with gold nanoparticles (black spots) [125]. Images adapted with permission from relevant references.

The largest subset of viruses are the bacteriophages—also known simply as ‘phages’—which only infect (and replicate within) bacterial cells. Most phages are known to be highly specialised in their ability to infect host bacteria, often affecting only single species or even single strains of bacteria. This narrow-spectrum activity makes them attractive as alternatives to conventional antibiotics, which are much less selective in their action and can, therefore, exert collateral AMR pressure on the beneficial and commensal microbiome in humans. A selection of therapeutically relevant phages is shown in Figure 6.

Phages and bacteria have been locked in an evolutionary battle for as long as they have co-existed. Both viruses and bacteria exhibit a high rate of genetic mutations, which in combination with their rapid reproduction/replication rate, large population sizes, and ability to exchange genetic material via horizontal gene transfer, allows them to evolve relatively quickly. Thus, the selection pressure exerted by phages on bacteria causes the latter to evolve features to resist phage infection, which in turn exerts a selection pressure on phages to evolve features to circumvent bacterial resistance.

From the standpoint of treating antibiotic-resistant bacterial infections, phage therapy offers tremendous therapeutic potential and is arguably the most viable alternative or adjunct therapy to antibiotics explored to date. Despite this, multiple challenges have so far prevented phage therapy from becoming a mainstay treatment in the clinic in the Western world—most notably issues with regulation and licensing, as well as production, equity, logistical barriers, and their potential role in further spreading antibiotic resistance genes.

There exist also viruses which infect other viruses or fungi, which are called virophages and mycoviruses, respectively; for clarity, ‘mycophage’ is a term typically used to refer to a subset of bacteriophages which infect mycobacteria. Their therapeutic potential for treating drug-resistant viral and fungal infections is much more limited than that of bacteriophages for treatment of bacterial infections. This is primarily due to the very narrow host range of virophages, and the restricted propagation mechanisms of mycoviruses, which usually lack an extracellular phase and hence suffer from inefficient transmission.

### 3.1. Classification

The incredible genetic variety of phages has necessitated the use of several concurrent forms of classification—phages may be classified in terms of morphology, genetic information, or their replication mechanism, among other features.

#### 3.1.1. Morphology and Genetic Information

**Figure 7:**
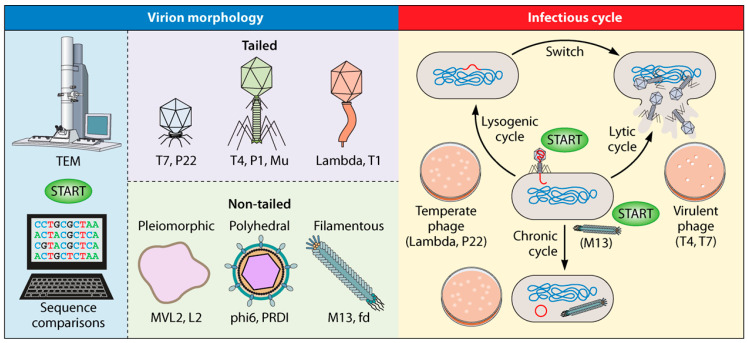
Illustration of phage classification by morphology and infectious cycle, adapted with permission from reference [128].

By far the most common phage morphology (shared by some 96% of all phages studied to date) [126] is that of the tailed phage, in which the virion (complete phage particle) consists of an icosahedral ‘head’ (or capsid) that contains its genetic material, attached to a tail which displays receptor-binding proteins that are used to recognise suitable host cells and transmit genetic material. They belong to the class *Caudoviricetes* and have double-stranded DNA genomes (dsDNA). Tailed phages may be further subdivided into podoviruses (short tails), myoviruses (long, contractile tails), and siphoviruses (long, non-contractile tails). The *Inoviridae* virus family are known as the filamentous phages, which have a single-stranded DNA genome (ssDNA), and typically a long, filament-like morphology (though some may be shorter and more rod-shaped). Non-tailed phage virions may be icosahedral (e.g., *Corticoviridae* (dsDNA), *Tectiviridae* (dsDNA), *Microviridae* (ssDNA)) or spherical (e.g., *Cystoviridae* (dsRNA), *Leviviridae* (ssRNA)); virophages in the class *Virophaviricetes* (dsDNA) also adopt an icosahedral morphology without a tail. Mycoviruses (predominantly dsRNA or ssRNA) may have a range of morphologies, from having no capsid at all (*Narnaviridae*), to having as many as four distinct virions (each carrying distinct parts of the mycovirus’ genome, such as *Chrysoviridae*) [127]. Selected examples of phage morphologies are summarised in Figure 7.

#### 3.1.2. Mechanisms of Replication

Perhaps of most importance for antibacterial applications is the type of replication cycle a phage undergoes. Phage replication cycles begin with the attachment of the phage virion to receptors on the surface of a susceptible bacterial host cell, whereupon it penetrates the bacterium’s cell wall/membrane to inject the phage genome into the host. After infection, the phage initiates the breakdown of the host genetic material, and the host cell begins to produce components to assemble new phage virions. This eventually culminates in the lysis (and therefore death) of the host cell, releasing the new virions into the extracellular environment, where they are free to propagate and to infect surrounding bacterial cells. This is known as the ‘lytic’ pathway, and is the preferred mechanism for antibacterial applications as the rapid propagation of the phages through the pathogenic bacterial population can quickly overwhelm an infection. Bacteriophages which replicate exclusively through the lytic pathway are known as ‘virulent’ phages.

Alternatively, some phages may follow the ‘lysogenic’ pathway, where the injected phage genome instead becomes integrated into the bacterial genome, or persists within the cell as a plasmid-like structure called an ‘episome’. The resulting bacterial host is called a lysogen, and the incorporated phage sequence is referred to as a ‘prophage’. As the lysogen reproduces, the population of bacteria containing the prophage grows. Occasionally, in response to external stressors, the dormant prophage may reactivate and proceed to replicate through the lytic pathway. Phages which undergo both lysogenic and lytic processes are termed ‘temperate’. The lysogenic and lytic pathways are illustrated in Figure 7.

Unlike virulent or temperate phages, filamentous phages do not follow the lytic or lysogenic cycles. They may replicate as an episome or by integrating into the host genome, where the host cell continuously produces virions which are extruded through the host’s cell membrane without undergoing lysis. This may slow down the replication rate of the host cell but does not typically kill it.

**Figure 8:**
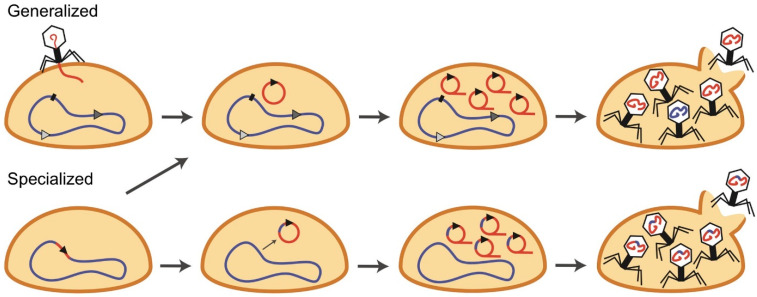
Schematic diagram illustrating the phage-mediated gene transfer mechanisms of generalised and specialised transduction. Adapted with permission from reference [130].

Another important concept is phage-mediated gene transfer between bacteria, also known as ‘transduction’ (Figure 8). In addition to conjugation and transformation, transduction is one of the ways in which horizontal gene transfer occurs between bacteria (and is therefore an important mechanism for the propagation of antibiotic resistance genes). Transduction can be generalised or specialised, occurring via the lytic or lysogenic pathways respectively. Generalised transduction happens when random fragments of bacterial genetic material are erroneously packaged into new virions during replication.

Specialised transduction, on the other hand, occurs when a prophage sequence is excised from the bacterial genome prior to replication, and may erroneously include bacterial genetic material at either end of the prophage sequence, which is then propagated to other bacterial cells along with the original phage genome. Where this process results in alterations to the host bacterium’s phenotype, it is called ‘phage conversion’. Phage conversion can result in host bacteria acquiring traits which drastically increase their virulence and pathogenicity. A notable example of this phenomenon is cholera, which is caused by the cholera toxin produced by strains of *Vibrio cholerae* infected with the filamentous CTXφ phage [129].

The use of temperate phages and filamentous phages for phage therapy applications is therefore less desirable, primarily due to their inability to rapidly and predictably deplete the pathogenic bacterial population, and also because of the possibility of introducing additional pathogenicity.

**Figure 9:**
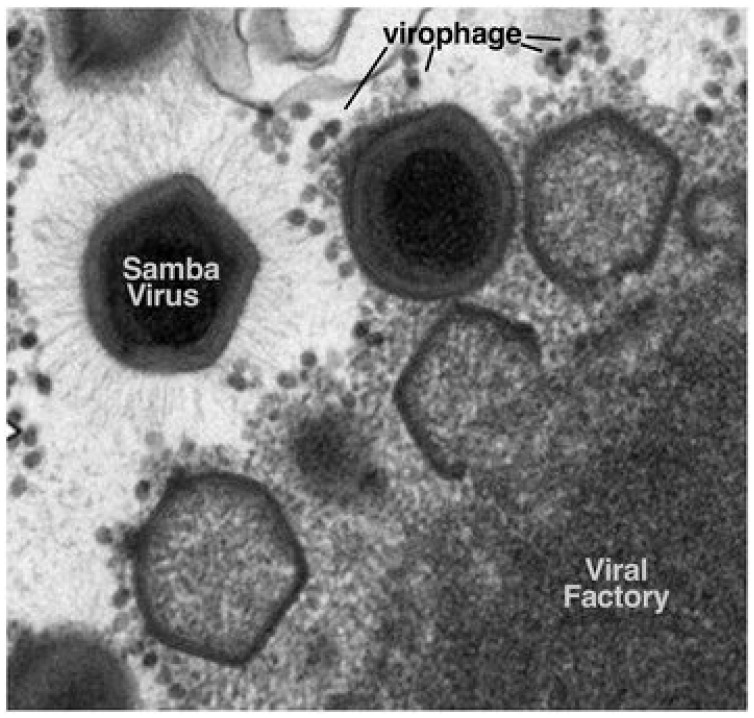
Scanning electron microscopy image showing Samba virus particles and viral factory, and associated parasitic Rio Negro virophages, within a host amoeba (*Acanthamoeba castellanii*). Adapted from reference [131].

The virophage replication cycle also occurs within a host cell—but, unlike bacteriophages, virophages are unable to exploit the host cell to produce virophage progeny directly. Instead, the virophage only replicates when the host cell is co-infected with a second virus (a giant virus). Specifically, when a giant virus infects a host cell (typically an amoeba or algal cell), it re-assembles some of the host cell contents into what is known as a ‘viroplasm’ or virus factory—the purpose of which, as the name implies, is to replicate the virus. Virophages target the viroplasm assembled by a giant virus and effectively parasitise it to produce virophage progeny (for instance, as shown in Figure 9). In many cases, this has the effect of significant inhibition in the replication of the giant virus (a decrease in virulence). Analogously to bacteriophages, virophages can integrate into the genome of both the giant virus and the host cell as provirophages to proliferate vertically.

Unlike bacteriophages or virophages, mycoviruses typically do not produce free virions which are capable of infecting fungal cells through extracellular transmission (with very few known exceptions [132]). Rather, mycoviruses are thought to be transmitted primarily through the cytoplasm—either vertically (by the formation of spores) or horizontally (through the fusion of cells in hyphae). A further barrier to efficient transmission of mycoviruses is that hyphal fusion between two individual fungi may not be successful if the fungi are not genetically compatible—where incompatibility occurs, a defensive process is initiated that triggers programmed cell death of the hyphae, thus preventing transmission of mycoviruses. Incompatibility may arise even between individual fungi of the same strain, which severely curtails the rate and extent to which mycoviruses can spread, and by extension their therapeutic potential.

### 3.2. Antimicrobial Therapeutic Applications

#### 3.2.1. Bacteriophages

The discovery of bacteriophages in the 1910s slightly predates the widespread use of conventional antibiotics spurred on by the discovery of penicillin in 1928, though research in the field of antibiotic development was already in progress from the late 19th century. Due to a combination of inconsistent results, practical limitations, and the socioeconomic landscape at the time, the initial research interest in phage therapy rapidly waned in favour of antibiotics in the West. In the Soviet Union, however, bacteriophages remained the subject of intense study for several decades, resulting in the adoption of their use therapeutically—including on a large scale for the treatment and prevention of bacterial infections in soldiers. Since the emergence of AMR and the increasing awareness of the need for alternatives to antibiotics, particularly in the last couple of decades, phage therapy has enjoyed a resurgence in both research intensity and clinical use.

Though bacteria can evolve resistance towards phages, phages can co-evolve allowing them to potentially counteract such emerging resistance phenotypes. One mechanism through which bacteria may develop resistance towards phages is through CRISPR-Cas adaptive immune systems, which can acquire fragments from the phage genome and subsequently use them to detect and destroy nucleic acid material from that type of phage [133].

Furthermore, resistance to bacteriophages may carry substantial fitness costs to the bacteria [134], including reduced virulence, impaired biofilm formation, or increased susceptibility to antibiotics, creating evolutionary trade-offs that may be therapeutically exploited. In some cases, phages may act synergistically with antibiotics, positioning them as an excellent adjunct therapy to conventional antibiotics.

Arriving at a therapeutic phage formulation to treat a particular infection, however, is not a trivial process. Phages are found wherever bacteria reside—in the case of bacteria which are pathogenic to humans, samples may be obtained from sources including patients (for instance in wounds or sputum) or from the environment, for example in medical waste streams or sewage. Such samples contain myriad different phages, so phages which are active against a particular type of bacterium must first be found and isolated (and subsequently, characterised).

To do this, samples are mixed with a suitable bacterial host (or multiple hosts), which determines the type of phages that can be isolated. During incubation, if any compatible phages are present, they will propagate and increase in concentration. After incubation, the bacteria and sample debris are removed using filtration or centrifugation, and any phages which are identified in the supernatant are then characterised to determine if they exhibit properties which are desirable for phage therapy [135]. In the ideal case, for a particular pathogenic bacterial species, phages will be identified which have: (1) broad-spectrum activity with respect to the strains of that species, to maximise therapeutic potential; (2) narrow-spectrum activity with respect to other bacterial species, to avoid elimination of non-pathogenic bacteria; (3) high virulence and low lysogenic potential (that is, they should propagate faster than the pathogenic bacteria and exclusively through the lytic pathway) to ensure their ability to overwhelm the bacterial infection and minimise the chance of gene transfer that may lead to further resistance.

Of course, in vitro measures of these factors do not always reflect the in vivo performance of phages, and pathogenic bacterial infections in humans do not necessarily occur as a result of a single species of bacteria. Hence, for therapeutic applications, it is more typical to administer a phage ‘cocktail’, or a mixture of phages that in sum have a broader host range than any of the component phages alone. In some countries, such as Georgia and Russia, phage cocktails are readily available over the counter—for instance, Pyophage, which is a liquid preparation of phages targeting *Staphylococcus*, *Streptococcus*, *Proteus*, *E. coli*, *P. aeruginosa*, and *Klebsiella* (Georgia only) [136]. However, such products are complex, and their composition may change due to evolutionary pressure over the course of serial propagation across decades—a property which many regulating agencies find unpalatable and which has thus far hindered licensing elsewhere. In addition, high-quality, modern clinical trials using one-size-fits-all phage formulations have thus far been limited in number and have yielded mixed efficacy results [137].

Poland, which too has an extensive history in phage research and therapeutic use, is home to Europe’s first Phage Therapy Unit, where phage therapy is more tightly regulated and still considered experimental [138]. Indeed, in many countries where phage therapy is permitted, it is primarily administered on compassionate use grounds—that is, as a last resort on a case-by-case basis—or as part of clinical trials. In Belgium, since 2018, phage therapy is regulated as a magistral preparation [139], whereby pharmacists may prepare custom phage therapy formulations for individuals as prescribed by their doctor and in accordance with Belgian Pharmacopoeia standards, without the need for market authorisation.

Custom phage preparations can be used in cases where cultures from a patient have identified specific infecting bacterial species for which effective phages are already known, but this requires access to extensive phage libraries from which appropriate phages may be sourced. The George Eliava Institute of Bacteriophages, Microbiology and Virology in Georgia hosts the largest collection of bacteriophages—currently totalling over 1000 phage species; multiple citizen science projects also exist which invite members of the public to submit environmental samples to phage banks around the world in search of undiscovered phages that may be of therapeutic interest [140].

There are many case reports of patients with recurrent antibiotic-resistant infections for which custom phage therapy has resulted in successful treatment and remission [141,142], though there are also some in which unfortunately the patients went on to develop resistance to phage therapy [143,144]. For example, data from the Institute of Immunology and Experimental Therapy in Poland indicate that up to 28.6% of patients with antibiotic-resistant *E. coli* infections developed resistance to all *E.coli*-specific phages in their collection [145]; in such cases, combination therapy using conventional antibiotics in synergy with phages may offer better outcomes. It is possible to alter the performance of phages using training (repeated exposure to bacterial cultures to drive coevolution) and genetic engineering approaches, for instance to lower the rate at which bacteria develop phage resistance [146,147], or to expand host range and evade host immunity [148,149,150]

A recent retrospective observational study [151] details the clinical outcomes of 100 patients treated with personalised phage therapy (with and without concomitant antibiotics, and in some cases with phage training) by a Belgian consortium across 12 countries between 2008 and 2022. Interestingly, the findings indicate a 70% higher probability of eradication when treated with a combination of phages and antibiotics, relative to phages without the administration of concomitant antibiotics. There is some indication in the study that trade-off effects from *P. aeruginosa* populations developing resistance to phages may decrease their virulence (2 of 16 cases with sufficient samples) and even re-sensitise them to antibiotics (of the fluoroquinolone class) to which they were previously resistant (1 of 16 cases). This finding is consistent with other studies which also reported similar re-sensitisation in bacteria under phage pressure [152], offering a promising avenue to combat antibiotic resistance. Moreover, only seven non-serious adverse reactions thought to be related to phage therapy were reported, supporting the notion that phage therapy is safe and well-tolerated.

There are several proposed mechanisms behind phage-antibiotic synergy (PAS) relevant to therapeutic lytic phages, major modes are summarised here but are explored in detail elsewhere [153]. Firstly, the presence of sub-inhibitory amounts of some antibiotics may elicit a stress response in bacteria, which can cause morphological changes, for instance cell elongation or cell clumping. Elongated cells have a higher surface area available for phage adsorption, as well as a higher number of viral factories and larger volume per cell, enabling more phage virions to be produced before lysis occurs [153]. Cells that are clumped provide easy access to phage progeny to infect neighbouring cells. Additionally, phage-driven re-sensitisation to antibiotics may provide another synergistic pathway, whereby bacteria alter their cell receptors as an adaptation to avoid recognition by phages, but this may have evolutionary trade-offs (such as downregulated efflux or increased permeability) rendering them more vulnerable to the action of antibiotics.

Conversely, antagonistic interactions between phages and antibiotics are also possible, but their mechanisms are less well understood. Antagonism may be mediated by antibiotics which target bacterial cell processes that are required for phage virion production (e.g., protein synthesis) or those which upregulate adaptive immune systems (CRISPR-Cas) [153]; predation by phages may also upregulate the production of biofilms in bacterial populations which may make them less vulnerable to the action of antibiotics.

One important safety consideration in phage therapy relates to endotoxins, which are lipopolysaccharides (LPS) liberated from the outer membranes of Gram-negative bacteria during lysis. LPS are potent triggers of the innate immune system, and at high doses may induce severe inflammatory responses (e.g., septic shock, organ failure, and death). Endotoxin release is not unique to phage therapy, and also happens with antibiotic-mediated bacterial lysis. However, in phage therapy, extensive bacterial lysis may occur over shorter timescales and potentially exacerbate the inflammatory response. Additionally, phage preparations may themselves contain endotoxins as a byproduct of bacterial propagation during manufacturing, necessitating rigorous purification to adhere to strict regulatory limits on permissible endotoxin concentrations in therapeutic formulations.

Aside from their specificity, phages may offer another significant advantage over conventional antibiotics—namely, their ability to penetrate defensive bacterial biofilms. They accomplish this by producing enzymes which can break down the various components within the extracellular matrices of biofilms. Additionally, as phages diffuse through the biofilm matrix and infect the bacterial cells embedded within it, they proliferate via the lytic cycle thereby increasing their concentration within the biofilm. It is estimated that up to 80% of chronic bacterial infections seen in humans arise from biofilm-producing bacteria [154]; therefore, the ability to penetrate and disrupt biofilms is a crucial property for more effective treatment. Here too, combined treatments using antibiotics and phages or phage-derived enzymes may offer additive advantages over phage-only or antibiotic only therapy [155,156]. However, this is not always the case—there is evidence in the literature that some filamentous bacteriophages (*P. aeruginosa* phages Pf, and *E. coli* phages Fd) may promote and support biofilm formation [157].

There may also be therapeutic potential in bacteriophages as adjunct or alternative therapies for treating viral and fungal infections [158], through immunomodulatory action or other mechanisms including inhibition of fungal metabolic processes [159].

Phage therapy in animals has been studied and applied in veterinary medicine both in livestock and companion animals, and the key findings are summarised extensively elsewhere [160,161,162]. The first commercially available phage preparation for aquaculture (CUSTUS^®^_YRS_) was released in 2018, intended for use as a biocontrol agent to prevent yersinosis (caused by *Yersinia ruckeri*) in farmed salmon. Other examples of commercially available phage products for veterinary use include SalmoFree^®^ and Bafasal^®^ (orally administered phage cocktails against *Salmonella*) [163,164], and Finalyse^TM^ (a topically applied phage formulation for cattle against *E. coli*) [165]. Aside from direct therapeutic uses, phages may potentially be used alone or in conjunction with disinfectants to disinfect hard surfaces, as a recent study has demonstrated [166].

#### 3.2.2. Virophages and Mycoviruses

The therapeutic potential of virophages and mycoviruses (for anti-viral and anti-fungal applications, respectively) is not well-explored, certainly relative to bacteriophages. To date, there exists only limited evidence concerning the potential pathogenicity of giant viruses in humans [167,168], and no virophages are known which parasitise common pathogenic viruses. Little is known so far about the mechanisms which govern virophage, giant virus, and host cell interactions—including their entry into the host cell, and the extent to which virophage co-infection can decrease the viral load of giant viruses within the host. Accordingly, the use of virophages in antiviral therapy remains speculative, with no experimental studies to date, though recent reviews have noted the need to better understand their mechanisms for potential therapeutic applications in the future [169,170]. In order for virophages to become realistic antiviral agents, not only must a thorough mechanistic understanding of their function first be developed, but it must also be established whether any virophages may intersect with human pathogenic viruses (as well as rigorous subsequent safety testing in mammalian hosts).

The prospects of mycoviral therapy to combat fungal infections are somewhat more promising. The programmed cell death process in fungi may be circumvented to increase efficacy of mycovirus transmission, which would make mycoviruses much more attractive as potential anti-fungal therapeutics. A recent study found that mycovirus transmission between incompatible strains of *S. sclerotiorum* (a pathogenic plant fungus) was significantly enhanced by treatment with proline—a naturally occurring amino acid. Specifically, proline was shown to downregulate the programmed cell death response and thus increase the rate of hyphal fusion between the strains. The group demonstrated this concept using field experiments, which showed 41.9% reduction in disease incidence in rapeseed plants treated with mycovirus and proline, compared to 26.9% decrease for mycovirus alone, relative to untreated plants [171]. It is possible that similar, as yet unknown, mechanisms may be available for exploitation to combat fungi which are pathogenic to humans.

It has also been hypothesised recently that the existing diversity of mycoviruses cannot be fully accounted for solely by intracellular transmission. Additional mechanisms of horizontal transmission have been proposed and are the subject of ongoing investigations—namely, vector and plant mediated transmission routes [172], though they are perhaps of less direct relevance to pharmaceutical applications. It does highlight that our current understanding of the transmission mechanisms of mycoviruses is by no means complete, and it is, therefore, possible that other novel avenues to enhance their transmissibility for therapeutic purposes will be found.

A well-established example of effective mycovirus-mediated fungal infection control is chestnut blight, caused by *Cryphonectria parasitica*. Chestnut blight infects the bark of chestnut trees, disrupting the flow of water and nutrients through the tree, and eventually causes death. The mycovirus *Cryphonectria hypovirus 1* infects *C. parasitica*, whereupon it induces changes including reduced fungal growth, sporulation, and pathogenicity [173].

Many species of mycovirus that can downregulate the virulence of fungal species have been identified, though efforts to use such mycoviruses to control infections to date have, by and large, focused on fungi affecting plants. Examples of mycoviruses which affect the phenotype and virulence of fungi that are pathogenic to humans are known, but their therapeutic potential has seldom been explored experimentally. In one study in mice, exploring the effect of mycovirus infection on the phenotype and virulence of *A. fumigatus* [174], it was found that *Chrysovirus* (AfuCV) infected *A. fumigatus* exhibited phenotypic changes and slower growth rate, but no significant difference in virulence (assessed by fungal burden in mouse lungs) was found. Conversely, a later study on the pathogenicity of *A. fumigatus* in moths found that infection with an uncharacterised mycovirus (A78) induced hypervirulence traits in the fungus [175], including increased growth rate.

More recently, significantly reduced virulence and lung fungal burden was found in mice pulmonary aspergillosis induced with *Chrysovirus* (AfuCV41362A) infected *A. fumigatus*, relative to virus-free *A. fumigatus* [176]. It is clear from such mixed results that the interplay between fungi and mycoviruses is complicated, and thus necessitates further study to elucidate the mechanisms which relate mycovirus infection to fungal pathogenicity.

#### 3.2.3. Parasite-Associated Viruses

The concept of exploiting viruses and their properties for the treatment of endoparasitic infections has been around for some time [177], with discussion focusing primarily on viruses infecting protozoan parasites [178] which represent many of the most prevalent and clinically significant human parasitic pathogens. While studies concerned with the detection and characterisation of viruses from clinical isolates of parasites are relatively abundant and span multiple decades, the number of experimental studies directly exploring the effect of such viruses on the parasites remains comparatively modest. For example, viruses associated with *Plasmodium* species [179] are known, but so far, their effect on the pathogenicity of the parasite has not been clearly established yet.

Leishmania RNA viruses (LRV 1 and 2) which infect *Leishmania* species known to be pathogenic to humans have been more extensively studied. An in vitro study on the infectivity and immunomodulatory effects of LRV-1 demonstrated that virus-positive parasites exhibit increased infectivity in human macrophages and induce a pro-inflammatory cytokine profile, characterised by elevated tumour necrosis factor α and interleukin 1β production [180]. These findings suggest that it may be possible to achieve improved therapeutic outcomes by applying antiviral therapy to target the LRV virus and mitigate virus-associated enhancement of parasite virulence. In support of this concept, a recent study identified two candidate nucleoside analogue drugs capable of reducing LRV-1 viral load; however, the serum concentrations of drug required to achieve meaningful viral load suppression were found to be higher than those practically achievable in animal models, indicating that antivirals with substantially greater potency would be needed for therapeutic translation [181].

Parasite-associated viruses have also been shown to modulate host immune responses in other protozoan infections. In *Trichomonas vaginalis*, the presence of Trichomonas virus is sensed by Toll-like receptor 3, which triggers an inflammatory response. This response was shown to be exacerbated by metronidazole treatment, which reduces parasite burden, but thereby releases more viral particles which are then sensed by the immune system [182]. This effect does not appear to translate into clinical outcomes; however, as a recent study found no significant difference between clinical symptoms, infection recurrence, or resistance towards metronidazole in virus-free archival clinical isolates of *T. vaginalis* compared to those which were positive for Trichomonasvirus [183].

In contrast to the above examples, parasite-associated viruses may also have a detrimental effect on parasite pathogenicity. For example, Giardiavirus, which infects *Giardia duodenalis*, appears to decrease the pathogenicity of *Giardia*—showing decreased disease severity in mice compared with virus-free *Giardia* [184]. Such observations raise the possibility that therapeutic administration of viruses may provide a pathway to suppress parasite pathogenicity, which may be especially useful for managing drug-resistant infections.

Viral sequences have been identified in various pathogenic helminth species, but there are no experimental studies to date on their effect on the pathogenicity and phenotype of helminths. A recent study identified 91 viruses in 41 species of human and animal parasitic nematodes. Nematode-associated viruses BMRV1 (which infects *Brugia malayi*) and OVRV1 (which infects *Onchocerca volvulus*) were shown to elicit an antibody response in vertebrates [185] providing evidence that such viruses are exposed to the host immune system. This observation is analogous to the findings described above for Trichomonasvirus and LRV- 1 and suggests that parasite-associated viruses may have the capacity to influence host-parasite immune interactions.

Insect-specific virus infections in ectoparasites may affect their ability to act as vectors for other infectious diseases, for example cell-fusing agent virus which infects mosquitoes and reduces their ability to transmit Dengue and Zika viruses [186]. While virus-based control strategies are unlikely to function as direct in-host treatments (as delivery to external parasites would be impracticable), they may provide a powerful, underexplored means of suppressing parasite-mediated transmission of infectious diseases at the population level.

### 3.3. Pharmaceutical Formulation of Phages

For any phage therapy to be effective, the phages must be viable when they are delivered to the patient. Hence, formulation approaches must consider phage stability at the point of formulation (such as by exposure to excipients, solvents, or processing conditions) as well as during transport or storage. Formulations must also conform to the requirements of the routes of administration for which they are intended, and to facilitate product licensing, should be fabricated in compliance with Good Manufacturing Practice (GMP) and rigorously tested through clinical trials; this latter aspect is particularly challenging for phage formulations, not least because phage mixtures must be individualised for each patient for optimum therapeutic outcomes.

#### 3.3.1. Phage Interaction with Host Immune System

While detailed discussions of the interactions between phages and the human immune system may be found elsewhere and are beyond the scope of this review [187,188], awareness of such interactions is crucial with respect to formulation of phage therapeutics. Though phages do not directly infect human cells, the human immune system is capable of recognising and reacting to their presence. Given the ubiquity of phages in the environment, and their widespread presence within the human body, endogenous phages typically do not elicit a strong inflammatory response under homeostatic conditions. As noted previously, in certain contexts, phages may be able to modulate the immune response of the host toward pathogens, which is advantageous because it helps to increase pathogen clearance and lead to improved therapeutic outcomes.

However, in therapeutic applications where large concentrations of exogenous phages are introduced, there is potential for activation of both innate and adaptive immune mechanisms. This may occur indirectly, for example, through inflammatory reactions to endotoxins released as a result of large-scale lysis of susceptible bacteria. Direct immune recognition of phages is also possible, including clearance by phagocytic cells and interactions with components of the complement system. The intensity of such reactions is variable depending on phage type and administration route and generally tend to be most intense for intravenously (IV) administered phages. While IV administered phages are the most bioavailable, they are particularly vulnerable to clearance by immune cells in the liver and spleen, resulting in their rapid removal from the bloodstream (and hence, potentially necessitating higher or repeated dosing to achieve therapeutic effects). Repeat dosing may, in turn, stimulate the adaptive immune system into producing phage-specific antibodies, which can neutralise the phages and diminish their therapeutic effectiveness [189]. Here particularly, strategies, such as encapsulation in nanoparticles, may offer significant improvements over currently used phage formulations by shielding phages from the immune system.

Overall, host–phage immune interactions represent both a barrier and a potential therapeutic asset. While immune clearance limits systemic persistence, immunomodulatory properties and the possibility of synergistic interactions with host defences offer opportunities to enhance treatment outcomes in certain contexts. Continued characterisation of these interactions, together with advances in formulation, will be essential for optimising the pharmacokinetics and clinical performance of phage-based therapies.

#### 3.3.2. Phage Production

Following isolation, phages undergo extensive characterisation to determine if the phage has suitable properties for medical applications. As described earlier, this includes the host range, lysogenic potential, and virulence of the phage, alongside kinetic properties such as replication rate which are necessary to determine scale-up reaction parameters.

Large scale production of phages in bioreactors is possible in a variety of operation modes, most commonly in batch operation, but also in single and multi-stage continuous operation. These are described in detail elsewhere [190] but are summarised here in brief. In batch operation, host bacteria are cultivated in a bioreactor until a target cell density is reached, at which point phages are introduced at a defined multiplicity of infection (ratio of phage to bacteria). Rapid phage propagation and host cell lysis follow, after which phage progeny is harvested and separated from cellular debris. While this approach is operationally simple, it requires extensive cleaning between runs, leading to downtime, and is more susceptible to contamination and batch-to-batch variability.

Single-stage continuous operation is more complex and involves the continuous supply of host bacteria and simultaneous removal of phage progeny. Although this mode offers improved scalability and productivity, prolonged exposure of phages to their host can promote the rapid emergence of phage-resistant bacterial populations. Multi-stage continuous systems partially address this limitation by separating host proliferation from phage infection, typically by culturing bacteria in an upstream reactor prior to phage exposure.

For clinical applications, phage manufacture must additionally comply with GMP requirements, imposing stringent controls on upstream and downstream processing. This includes the use of well-characterised bacterial production strains, traceable raw materials, validated bioreactor cleaning procedures, and robust purification steps to remove host cell debris, endotoxins, and residual DNA. Batch production is currently more commonly adopted for GMP-compliant phage manufacture due to its relative simplicity and ease of validation, although continuous approaches remain of interest as regulatory frameworks and process control technologies mature.

#### 3.3.3. Phage Viability

While the stability of phages against environmental factors is variable between species, they are all composed of proteins which are crucial for their activity. Any conditions which may cause denaturation of these proteins (for instance high temperature, high acidity or alkalinity, mechanical stress, chemical reaction, or ionising radiation) can be expected to affect the activity of phages. A common method of measuring phage viability is by conducting a plaque assay—a susceptible host bacterial stock is mixed evenly with serial dilutions of phage sample and plated over solid agar in a petri dish [191]. During incubation, the bacteria proliferate evenly across the plate forming an opaque ‘lawn’; where a virulent phage is present, it infects the host bacterium and proliferates locally, generating a ‘plaque’ or bacteria-free region which appears clear. The plaques are then counted, and the concentration of viable phages in the original sample is expressed as plaque forming units per millilitre (PFU/mL, referred to as the titre). As phage titres required for therapeutic applications are generally very large, they are commonly expressed using the logarithmic scale.

#### 3.3.4. Routes of Administration and Dosage

Phages may be delivered by several routes, although the most frequently reported clinical routes are IV and topical. The choice of delivery route depends on the nature of the infection, and sometimes a combination of routes may be used.

IV administration is the preferred route for severe, systemic, or deep-tissue infections, as it has direct systemic availability, the broadest distribution in the body, and the most rapid onset of action. Formulations of phages intended for IV administration must comply with the general requirements of IV formulations (e.g., sterility), and most importantly, the total endotoxin amount administered must be below the acceptable level (which is defined by regulatory agencies in endotoxin units (EU) per kilogram of body weight per hour; for example, 5 EU/kg/hr as per the US Food and Drug Administration). This requirement limits the total dose and frequency of phage formulation that may be administered intravenously, a fact which must be weighed against the rapid immune clearance typically seen for IV administered phages. Repeated or long-term administration via IV may lead to decreased effectiveness over time due to antibody neutralisation.

Topical or local administration, on the other hand, is commonly used for infections that are superficial—for instance, as gels, creams, sprays, or dressings to treat skin wounds such as burns or ulcers, or outer ear infections. This type of application leads to high local concentrations of phages at the site of infection, with minimal systemic exposure (and therefore less risk of adaptive immune response) and is particularly useful for treating infections with a biofilm component.

Oral administration of phage formulations is typically limited to treatment of gastrointestinal infections and may necessitate the use of enteric coatings to preserve phage viability through the gastric environment. Absorption of phages through the GI tract is minimal and variable between individuals and is generally not considered suitable for treating systemic infections.

Less commonly, phages may be administered via inhalation, for example to treat lung infections associated with cystic fibrosis. As with topical administration, the advantage of this delivery route is local delivery of a high concentration of phages with low systemic exposure. However, the process of nebulisation may affect phage viability, and mucus and biofilm permeation may be limited. Finally, phages may be delivered directly into joints or the bladder for the treatment of prosthetic joint infections or urinary tract infections, respectively, although these routes are used infrequently and are inherently more invasive.

Unlike conventional antimicrobials, phages are self-amplifying (in the presence of susceptible host bacteria), and more vulnerable to immune clearance and environmental conditions. As a result, the most important outcome of administration is not the maintenance of steady plasma levels of phage within a therapeutic window, but rather the successful delivery of a sufficient number of infective virions to the site of infection (which can then go on to self-amplify). For this reason, dosing is typically empirical and informed by practical and regulatory constraints. Often, multiplicity of infection values are used to describe the ratio of phages to target bacterium, but for a variety of reasons [192], they may not always be a practical or informative measure in clinical settings.

#### 3.3.5. Phage Formulations

Detailed discussions of various phage formulation strategies and considerations are available elsewhere, [193,194,195] but are outlined in brief here.

Phages may be stored suspended in liquid, such as the Phago-Pyo cocktail which is supplied in 0.9% NaCl (saline), with a shelf life that is typically on the order of 1 to 2 years. A 2021 study investigated the stability of four different phages—Acibel004, PNM, 14-1, and ISP—stored in buffer and infusion solutions commonly used in medicine [196]. The findings indicate that for these four phages, the optimal storage medium was Dulbecco’s phosphate buffered saline (without Ca^2+^/Mg^2+^), resulting in around 1 log PFU loss over the course of 282 days, compared to the worst performing suspension agent (5% glucose), which immediately deactivated the PNM and ISP phages. Additionally, the initial phage concentration also appears to play a role in the retention of viability upon storage, with the higher concentration tested (9 log PFU) generally showing slower rates of viability loss over time. In general, such preparations are simple to prepare and convenient to use, but phages without additional stabilisation are vulnerable to degradation in response to environmental factors.

Protection of phages may be conferred by encapsulation into nanoparticles—for example, lipidic or polymeric nanoparticles. A recent study found that encapsulation of PEV2 and PEV40 phages into liposomes helped to stabilise them during nebulisation (for inhaled therapy applications), with titre loss of 0.07 log PFU and 0.4 log PFU, respectively, compared to 1.23 log PFU for free phages [197]. Another study encapsulated MRSA phages in chitosan (a biopolymer) and found drastically enhanced stability against pH extremes and high temperatures—for example, after 2 h incubation at 60 °C, the free phage viability decreased to ca. 7% of the initial viability, whereas encapsulated phages retained above 80% of the initial viability [198]. Interestingly, chitosan encapsulation also appeared to increase the stability of phages against pH extremes—at gastric pH (1.5), encapsulated phages retained ca. 90% viability as opposed to around 50% for free phages. This strategy may therefore be utilised for developing oral dosage forms of phages.

**Figure 10:**
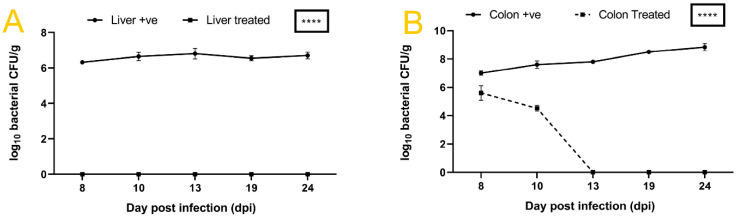
Graphs showing the bacterial load in the (**A**) livers and (**B**) colons of chickens infected with Salmonella paratyphi, untreated (+ve) versus treated with orally administered alginate beads loaded with phages. Adapted from reference [199]. **** indicates *p* < 0.0001 difference in bacterial load between treated and untreated birds.

A recent in vivo study explored the formulation of *Salmonella* phages into alginate beads, with several polymeric additives (polyvinyl acetate, polyvinylpyrrolidone) to protect against the gastric environment for oral phage delivery [199]. The study demonstrated the successful deployment of these formulated phage beads to treat *Salmonella* sp. infections in broiler chickens, with complete bacterial elimination in all tissues tested (including the colon, Figure 10). The ability of the formulation to deliver viable phages to the colon is notable, because free phages were found to be inactivated completely in a gastric simulation fluid.

Alternatively, suspensions of phages in solutions containing excipients, such as sugars, amino acids, or polymers (for bulking and/or stabilisation), may be processed by spray-drying or freeze-drying to produce solid forms. Encapsulating phages into solid matrices is advantageous because they can be better protected from environmental stressors, such as high temperature, ultraviolet radiation, mechanical stress, and dehydration. Spray-drying involves the atomisation of the liquid through a nozzle into a drying chamber full of hot gas, whereupon solvent is rapidly removed, producing homogeneous powders consisting of microparticles. Freeze-drying, on the other hand, utilises freezing followed by lowering of pressure to cause sublimation (and, therefore, dehydration). Freeze-drying is the preferred method for long term storage, in addition to freezing phages within their host bacteria. Both spray- and freeze-drying are already extensively used in industry, and, therefore, benefit from scalability and availability. Additionally, the lighter mass of dry phage formulations is cheaper to store and transport than liquid suspensions. However, an initial decrease in phage viability is often observed as a result of the stresses incurred during these processes (e.g., temperature and pH changes, ice crystal formation). For instance, freeze-drying of ISP phages under various conditions has been shown to lead to an immediate viability decrease (though the extent of this was highly dependent on the identity and concentration of stabilising excipients) [200].

In the second part of a buffer stability study [196], the stability of freeze-dried ISP phages (stabilised by different concentrations of sucrose or trehalose) after storage at 4 °C for 8 years was assessed. It was found that the initial loss of viability due to the freeze-drying process was 0.43–1.4 log PFU, and rehydrated samples after 8 years did not show any statistically significant decrease in viability in most cases. In another study by the same group, five phages (Acibel004, Acibel007, PNM, 14-1, and ISP) were mixed with 13 commercially available topical burn treatment products, including hydrogels [201]. The authors noted that due to the complex compositions of such products, it was difficult to determine which ingredients contributed most to the reduction in activity, but it appeared that products with antimicrobial components and high acidity (e.g., Bactroban and povidone-iodine) seemed to reduce phage viability most. In general, though compatibility appears to be highly variable by product and phage, most combinations tested appear to retain acceptable phage activity over at least 4 h.

**Figure 11:**
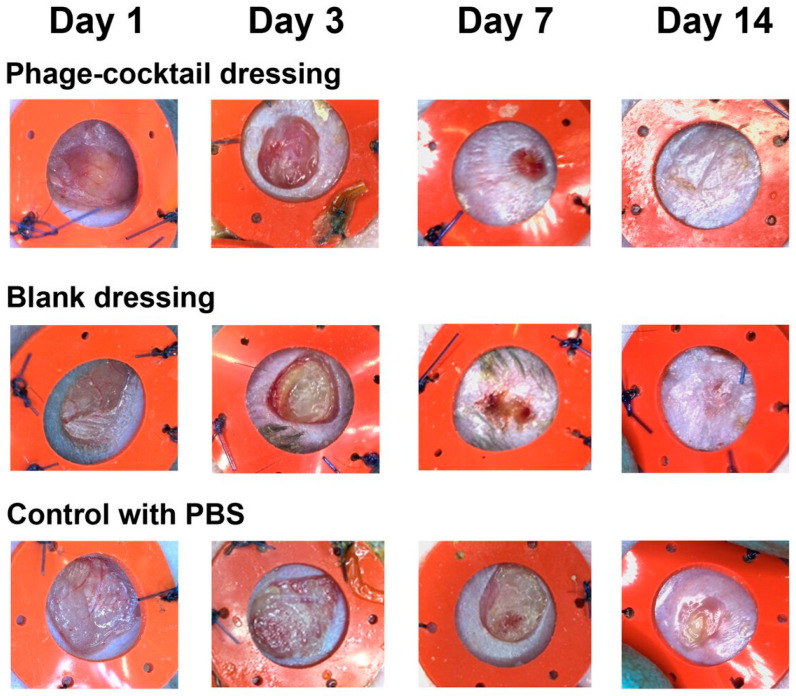
Photographs of wounds of animals in different treatment groups (phage dressing, blank dressing, and control) at different timepoints following injury. Adapted with permission from reference [201].

It is also possible to formulate phages directly into hydrogels to benefit from controlled release properties. For instance, a recently reported gelatin-based hydrogel impregnated with *P. aeruginosa* phages, where phage release is triggered in response to the presence of *P. aeruginosa* gelatinase enzymes. When the local concentration of bacteria is high, more enzymes are produced which cause increased hydrogel degradation and, therefore, higher amount of phage release [202]. In another study, the wound-healing ability of alginate hydrogel dressings containing various phages was tested in a full-thickness non-infected diabetic ulcer wound model [203]. It was shown that the presence of phages helped to aid wound healing (Figure 11, relative to blank dressing or saline only), possibly through modulatory effects on epithelial and immune cells.

Electrospinning is a production method which is commonly used in laboratory settings to make nanofibres and is increasingly explored as a method to stabilise phages for antibacterial wound dressing applications [204,205,206]. Briefly, a solution containing phages and polymer is pumped through an electrically charged needle, and the electrical potential difference between a collector and needle forces the solution to extrude into thin fibres. The resultant nanofibre mats have a high surface area and can release phages in a controlled manner as the fibres slowly disintegrate, while protecting the viability of the phages. Electrospraying is a similar process, which produces micro/nanoparticle powders instead of fibres.

Compared to spray-drying and freeze-drying, electrospinning and spraying are gentler and, therefore, also offer additional formulation flexibility, because it is possible to co-load other therapeutic agents (e.g., enzymes, antibiotics) which may not be stable to heat or ice crystal formation. Additionally, using a core-shell needle, it is possible to produce fibres with distinct core and shell compositions. This makes it possible, for example, to control release properties of phage by adjusting the shell composition (and hence, degradation speed). Recent work has produced core-shell fibres containing Neko phages [207]. The study showed that the fibres had good stability in storage at −20 °C, with no significant viability loss after storage for 6 months. However, significant viability loss was observed at storage at 4 °C and room temperature. and a range of release profiles from immediate to extended by varying the shell composition. Another recent study investigated the electrospinning of short-tailed (APTC-SL.1) and long-tailed (APTC-Efa.20) phages into core-shell fibres and found significant differences in the antibacterial efficacy of the phages after electrospinning [206]. The construct containing long-tailed phages exhibited no antibacterial effect, whereas the one containing short-tailed phages showed clear zones of lysis around the fibres after 24 h of incubation, demonstrating that phage morphology may also play a role in determining stability during formulation/processing.

A study encapsulating phages into electrosprayed particles demonstrated that this approach can also preserve phage viability while enabling the production of dry powder formulation with improved antibacterial performance (complete inhibition) relative to phage stock, and viability retention over 8 weeks in storage at −20 °C [207]. Another work comparing freeze drying, spray drying, and electrospraying on Felix 01 phages found the highest initial decrease in viability from freeze drying, but a slower rate of activity loss over 180 days of storage relative to spray dried and electrosprayed samples [208]. All three methods produced samples which had a higher degree of stability during storage than unencapsulated phage (ca. 0.18–0.49 log PFU versus 3.5 log PFU loss). At the end of the storage period, the viability of phages encapsulated using electrospraying and spray drying remained higher than that of freeze-dried phages, indicating that for short and intermediate storage terms these methods may be more suitable for preserving viability.

## 4. Conclusions, Barriers to Translation, and Future Outlook

As the antimicrobial resistance crisis continues to deepen, it is critical that emerging virus-based therapies do not repeat the shortcomings of the conventional antibiotic era. Decades of empirical prescribing and routine use of broad-spectrum antimicrobial agents has led to the current AMR landscape, with growing disease burden and grave concerns regarding our continued power to fight pathogenic microbes. With the increased interest in virus-based antimicrobial therapies, there is now an opportunity to move towards multi-pronged, individualised, and microbiome-friendly interventions to achieve better therapeutic outcomes and conserve the efficacy of critically important antimicrobial drugs for the future. Outside of their use for direct disease control via pathogen lysis as described in this review, there is also ample scope for the use of phages or viruses as targeted delivery vehicles, for example to deliver CRISPR-Cas systems for antimicrobial applications [209] and beyond [210].

Despite significant scientific progress and increasing clinical interest, a number of scientific and pharmaceutical challenges will need to be overcome in order to realise the potential of virus-based antimicrobial therapy. From a formulation perspective, strategies which cloak phages from immune recognition and rapid clearance are needed, particularly for intravenous delivery phage products. In parallel, formulation approaches should be explored which can enhance the bioavailability of phages/viruses by other delivery routes (e.g., oral, topical) as well as for improving the shelf stability and patient compliance. Beyond pharmacokinetics, a greater understanding of the nature of host immune responses to therapeutic viruses must be developed, as phages may exert immunomodulatory effects that may have ramifications for therapeutic efficiency as well as safety, particularly for repeated and long-term use.

Furthermore, it is essential to develop a deeper, mechanistic insight into virus-pathogen interactions, particularly to determine which facets of viral infection may modulate pathogenicity. Better understanding of the evolutionary interplay between phages and bacteria is critical, particularly in relation to phage-resistance and how it may lead to reduced pathogen virulence. Anticipating and exploiting such co-evolutionary interactions will be central to the rational design of virus-based therapies. Additional strategies which enhance the horizontal transmission of mycoviruses should be investigated to enhance their therapeutic potential. Research is needed to determine if virophages which parasitise human pathogens exist, or if it may be possible to exploit their mechanisms of action to engineer therapeutically relevant virophages. Phage engineering, for instance using CRISPR-Cas, offer additional opportunities to enhance therapeutic performance of phages, for example, by selectively removing virulence genes in the pathogen, or by producing biofilm-penetrating enzymes.

Continued expansion of phage discovery, characterisation, and cataloguing initiatives will enable the development of extensive viral libraries that are necessary for personalised therapy and scalable clinical deployment. As with conventional antibiotics, diagnostic infrastructure presents a major barrier to sustainable, individualised treatment. Limited and inequitable access to culture facilities constrains pathogen identification, while rapid phage susceptibility testing is not yet available. Current methods remain labour intensive and time consuming, so the development of high-throughput, automated methods which are capable of screening clinical samples against standardised phage libraries within clinically relevant timeliness is needed.

Regulatory divergence across countries is one of the central barriers to clinical deployment. Traditional drug approval frameworks are designed for well-defined, static products, whereas phages are evolving biologics whose composition may need to adapt over time. Belgium’s magistral preparation pathway remains the most progressive model, allowing bespoke phage preparations to be produced in pharmacies under GMP-like conditions without requiring full market authorisation. However, this approach has not yet been widely adopted elsewhere.

Access remains a critical concern, as phage matching, GMP production, and quality assurance can be costly relative to conventional antimicrobials. International collaboration and targeted funding will be essential to ensure equitable access, particularly in low- and middle-income countries, while technological maturation improves economic viability. Commercial incentives remain limited due to intellectual property uncertainty and unpredictable demand, reinforcing the need for coordinated public–private investment.

Clinical translation is complicated by the relative scarcity of large, standardised, and randomised controlled trials. Much of the current evidence base derives from compassionate use cases and heterogeneous study designs, underscoring the need for standardisation for comparability across trials. Moreover, combination therapy with antibiotics currently appears to yield the most reliable outcomes. Understanding how to optimally design and sequence phage–antibiotic regimens—including dosing intervals, synergistic pairings, and mitigation of antagonistic interactions—represents a key translational research priority.

Finally, societal and infrastructural factors will play a decisive role in determining the impact of therapeutic viruses. Shifting away from empirical antimicrobial use will require changes in clinical culture, alongside sustained education of healthcare professionals and patients, including addressing public perceptions and misconceptions surrounding the use of viruses as medicines. Importantly, robust antimicrobial stewardship is a prerequisite for success, particularly given that phage–antimicrobial combination therapies currently appear to offer the most consistent clinical benefit.

## Figures and Tables

**Table 3:** Selected pathogenic fungi and their corresponding priority level and AMR characteristics (WHO, 2022) [40].

Order	Family	Pathogen	Priority Level (WHO Rating, 2022) [40]	Resistance
*Tremellales*	*Cryptococcaceae*	*Cryptococcus neoformans*	Critical	Poorly understood; reduced susceptibility to fluconazole
*Eurotiales*	*Aspergillaceae*	*Aspergillus fumigatus*	Critical	Rising azole resistance
*Serinales*	*Metschnikowiaceae*	*Candida auris*	Critical	Fluconazole resistant; moderately resistant to amphotericin B
	*Debaryomycetaceae*	*Candida albicans*	Critical	Low, but increasing
		*Candida parapsilosis*	High	Moderate
		*Candida tropicalis*	High	Moderate resistance to azoles
*Saccharomycetales*	*Saccharomycetaceae*	*Nakaseomyces glabrata*	High	Rising azole and echinocandin resistance
*Hypocreales*	*Nectriaceae*	*Fusarium* spp.	High	High: resistant to most antifungals, particularly azoles
*Onygenales*	*Ajellomycetaceae*	*Histoplasma* spp.	High	Moderate

**Table 4:** Major antifungal classes, their mechanisms of action, typical spectra of activity, and dominant antifungal resistance mechanisms.

Antifungal Class	Mechanism of Action	Typical Spectrum	Representative Examples	Dominant AMR Mechanisms	AMR Relevance/Notes
Echinocandins	Inhibit β-1,3-D-glucan synthase: impaired cell wall synthesis	*Candida* spp., *Aspergillus* spp.	Caspofungin, Micafungin	FKS gene mutations (glucan synthase alteration)	Low resistance overall; emerging in Candida glabrata
Azoles	Inhibit lanosterol 14α-demethylase (ERG11): disrupted ergosterol synthesis	Broad (*Candida*, *Aspergillus*, *Cryptococcus*)	Fluconazole, Itraconazole, Voriconazole	Target mutation; target overexpression; efflux pumps	Major driver of clinical antifungal resistance
Polyenes	Bind ergosterol: membrane pore formation	Broad	Amphotericin B	Reduced ergosterol content; membrane alterations	Resistance rare but toxicity limits use
Allylamines	Inhibit squalene epoxidase: ergosterol depletion	*Dermatophytes*	Terbinafine	Target mutations; altered sterol pathways	Resistance increasing in dermatophytes
Pyrimidine analogues	Inhibit DNA/RNA synthesis after intracellular conversion	Narrow (*Candida*, *Cryptococcus*)	Flucytosine	Loss of drug uptake; metabolic bypass	Rapid resistance in monotherapy
Orotomides	Inhibit dihydroorotate dehydrogenase: pyrimidine synthesis blockade	*Aspergillus* spp.	Olorofim	Target-site mutations (emerging)	No activity vs. Candida or Cryptococcus
Novel/emerging agents	Various (e.g., membrane phospholipids, β-glucan targeting)	Broad (experimental)	Mandimycin, Coniotins	Not yet defined	Early-stage; promising against MDR fungi

**Table 6:** Major antiviral classes, their mechanisms of action, typical spectra of activity, and dominant antiviral resistance mechanisms.

Antiviral Class	Mechanism of Action	Typical Spectrum	Representative Examples	Dominant Resistance Mechanisms	AMR Relevance/Notes
Nucleoside/nucleotide analogues	Incorporation into viral DNA/RNA: chain termination or polymerase inhibition	Broad (DNA & RNA viruses; virus-specific)	Acyclovir, Tenofovir, Sofosbuvir	Target-site mutations in viral polymerase; altered drug activation	Most widely used antivirals; resistance common in chronic infections
Non-nucleoside polymerase/ RT inhibitors	Allosteric inhibition of viral polymerase or reverse transcriptase	Narrow (virus-specific)	Etravirine, Nevirapine	Point mutations in polymerase binding pocket	Low barrier to resistance in monotherapy
Protease inhibitors	Inhibit viral protease: defective virion maturation	HIV, HCV	Ritonavir, Lopinavir, Simeprevir	Protease gene mutations; compensatory fitness mutations	Resistance reduced via combination therapy
Integrase inhibitors	Block integration of viral DNA into host genome	HIV	Dolutegravir, Raltegravir	Integrase target mutations	High genetic barrier; resistance increasing in poorly adherent populations
Entry/ fusion inhibitors	Prevent viral attachment or membrane fusion	HIV; limited others	Enfuvirtide, Fostemsavir	Envelope or receptor-binding protein mutations	Typically reserved for MDR infections
Capsid/ uncoating inhibitors	Disrupt capsid stability or uncoating	Influenza, emerging targets	Amantadine	Capsid protein mutations	Rapid resistance; limited modern use
Neuraminidase inhibitors	Inhibit viral release from host cells	Influenza A & B	Oseltamivir	Neuraminidase mutations	Seasonal resistance observed

**Table 7:** Major antiparasitic classes, their mechanisms of action, typical target organisms, and dominant resistance mechanisms.

Antiparasitic Class	Mechanism of Action	Typical Targets	Representative Examples	Dominant Resistance Mechanisms	AMR Relevance/Notes
Artemisinins	Heme-activated cleavage producing reactive radicals that damage parasite proteins and membranes	*Plasmodium* spp.	Artemisinin, artesunate	Delayed parasite clearance; altered stress response and metabolism	First-line malaria therapy; partial resistance emerging in Southeast Asia and Africa
Quinoline antimalarials	Inhibit heme detoxification by blocking hemozoin formation	*Plasmodium* spp.	Chloroquine, quinine	Reduced drug accumulation via transporter mutations	Widespread resistance limits use
Antifolate combinations	Inhibit folate biosynthesis	*Plasmodium* spp.	Sulfadoxine–pyrimethamine	Target enzyme mutations	Resistance common; largely obsolete for treatment
Nitroimidazoles	Redox activation within parasite: DNA damage and metabolic disruption	*Giardia*, *Entamoeba*, *Trichomonas*	Metronidazole	Reduced drug activation; altered redox enzymes	Increasing resistance reported in multiple protozoa
Antimonials	Disrupt parasite redox balance and energy metabolism	*Leishmania* spp.	Sodium stibogluconate	Reduced uptake; metabolic bypass	High treatment failure rates in endemic regions
Benzimidazoles	Bind β-tubulin, inhibiting microtubule polymerisation	Helminths	Albendazole, mebendazole	β-tubulin gene mutations	Major resistance issue in livestock and companion animals
Macrocyclic lactones	Activate glutamate-gated chloride channels causing paralysis	Helminths, arthropods	Ivermectin, moxidectin	Channel mutations; altered drug transport	Widespread resistance in veterinary settings
Neonicotinoids	Nicotinic acetylcholine receptor agonists	Insect ectoparasites	Imidacloprid	Target-site insensitivity	Reduced field efficacy increasingly reported
Pyrethroids	Prolong opening of voltage-gated sodium channels	Insect ectoparasites	Permethrin	Sodium channel mutations	Resistance common in fleas and ticks
Isoxazolines	Inhibit GABA- and glutamate-gated chloride channels	Fleas, ticks	Fluralaner	Emerging; mechanisms poorly characterised	Newer class; resistance surveillance ongoing

## Data Availability

No new data were created or analyzed in this study. Data sharing is not applicable to this article.
